# Impact of Including Korean Randomized Controlled Trials in Cochrane Reviews of Acupuncture

**DOI:** 10.1371/journal.pone.0047619

**Published:** 2012-10-11

**Authors:** Kun Hyung Kim, Jae Cheol Kong, Jun-Yong Choi, Tae-Young Choi, Byung-Cheul Shin, Steve McDonald, Myeong Soo Lee

**Affiliations:** 1 Department of Acupuncture and Moxibustion Medicine, Korean Medicine Hospital, Yangsan, South Korea; 2 Department of Rehabilitation Medicine, College of Korean Medicine, Wonkwang University, Iksan, South Korea; 3 Department of Korean Medical Science, School of Korean Medicine, Pusan National University, Yangsan, South Korea; 4 Medical Research Division, Korea Institute of Oriental Medicine, Daejeon, South Korea; 5 Department of Rehabilitation Medicine, School of Korean Medicine, Pusan National University, Yangsan, South Korea; 6 Australasian Cochrane Centre, School of Public Health and Preventive Medicine, Monash University, Melbourne, Victoria, Australia; University of Illinois-Chicago, United States of America

## Abstract

**Objective:**

Acupuncture is commonly practiced in Korea and is regularly evaluated in clinical trials. Although many Cochrane reviews of acupuncture include searches of both English and Chinese databases, there is no information on the value of searching Korean databases. This study aimed to investigate the impact of searching Korean databasesand journals for trials eligible for inclusion in existing Cochrane acupuncture reviews.

**Methods:**

We searched 12 Korean databases and seven Korean journals to identify randomised trials meeting the inclusion criteria for acupuncture reviews in the *Cochrane Database of Systematic Reviews*. We compared risk of bias assessments of the Korean trials with the trials included in the Cochrane acupuncture reviews. Where possible, we added data from the Korean trials to the existing meta-analyses in the relevant Cochrane review and conducted sensitivity analyses to test the robustness of the results.

**Results:**

Sixteen Korean trials (742 participants) met the inclusion criteria for eight Cochrane acupuncture reviews (125 trials; 13,041 participants). Inclusion of the Korean trials provided data for 20% of existing meta-analyses (24 out of 120). Inclusion of the Korean trials did not change the direction of effect in any of the existing meta-analyses. The effect size and heterogeneity remained mostly unchanged. In only one meta-analysis did the significance change. Compared to the studies included in the Cochrane acupuncture reviews, the risk of bias in the Korean trials was higher in terms of outcome assessor blinding and allocation concealment.

**Conclusions:**

Many Korean studies contributed additional data to the existing meta-analyses in Cochrane acupuncture reviews. Although inclusion of these studies did not alter the results of the meta-analyses, comprehensive searches of the literature are important to avoid potential language bias. The identification and inclusion of eligible Korean trials should be considered for reviews of acupuncture.

## Background

Systematic reviews and meta-analyses of the best available evidence can inform decision-making in clinical practice, guide further research, and lead to the efficient allocation of resources [1]. The *Cochrane Database of Systematic Reviews* (*CDSR*) is regarded as a significant and reliable resource of systematic reviews of the effects of a broad range of healthcare interventions in both conventional and complementary medicine. The reputation of Cochrane reviews is based on their comprehensive search strategies, periodical updates and rigorous analytic methods [2].

Acupuncture is a therapeutic intervention that has traditionally been used in East Asian regions, such as China, Korea, Japan, and Vietnam, and one that more recently, has been increasingly accepted and popularized in Western societies. Many controlled clinical trials of acupuncture are widely available in medical databases (e.g., MEDLINE). At the same time, there are also many studies that are indexed in less widely available local databases that include languages other than English as the primary language [3], [4], [5], [6].

When preparing Cochrane reviews, it is strongly recommended that review authorssearch at least three databases that use English as the principal language (i.e., EMBASE, MEDLINE and CENTRAL) and conduct extensive literature searches that cover all relevant languages to avoid publication, language, and citation biases [7]. An empirical study revealed that language bias derived from language-restricted search strategies, or from ignorance of certain databases that employ languages other than English, has been known to significantly affect the results of systematic reviews in complementary and alternative medicine (CAM) [8]. This study showed that systematic reviews of CAM resulted in 63% smaller effect estimates when only English studies were included compared to those without any restriction on thelanguage of included studies. However, in that study, most of the CAM trials in languages other than English (LOE) were published in European countries that had evaluated CAM interventions other than acupuncture. Thus, the impact of language bias due to the omission of papers from Asian databases when evaluating the evidence about effects of acupuncture still remains largely unknown.

**Table 1:** Search terms used and journals searched.

Search terms used	
Acupuncture related	Acupuncture OR acupressure OR acupoint OR meridian OR acup*
Design related	Random OR control OR group OR divide
Journals searched	Journal of Korean Acupuncture and Moxibustion Society
	Korean Journal of Acupuncture (formerly the Journal of Korean AM-Meridian & Pointology Society)
	Journal of Pharmacopuncture
	Journal of Oriental Rehabilitation Medicine
	Journal of Korea CHUNA Manual Medicine for Spine & Nerves
	Journal of Korean Oriental Medicine
	Journal of Korean Oriental Internal Medicine

In Cochrane acupuncture reviews, the decision to search databases of languages other than English seems to depend, at least partly, on individual review authors and the topic-related Cochrane Review Groups (CRGs). As a result, the search strategies of many Cochrane reviews of acupuncture are characterized by considerable heterogeneity [9]. A previous study found that the number of databases searched varied among Cochrane acupuncture reviews, with only two out of ten reviews searching Chinese databases [4]. Another study revealed that 26 out of 65 Cochrane acupuncture reviews and protocols searched Chinese language databases [9]. Both studies emphasized the inclusion of Chinese databases to prevent bias associated with the exclusion of controlled trials reported in languages other than English.

**Figure 1:**
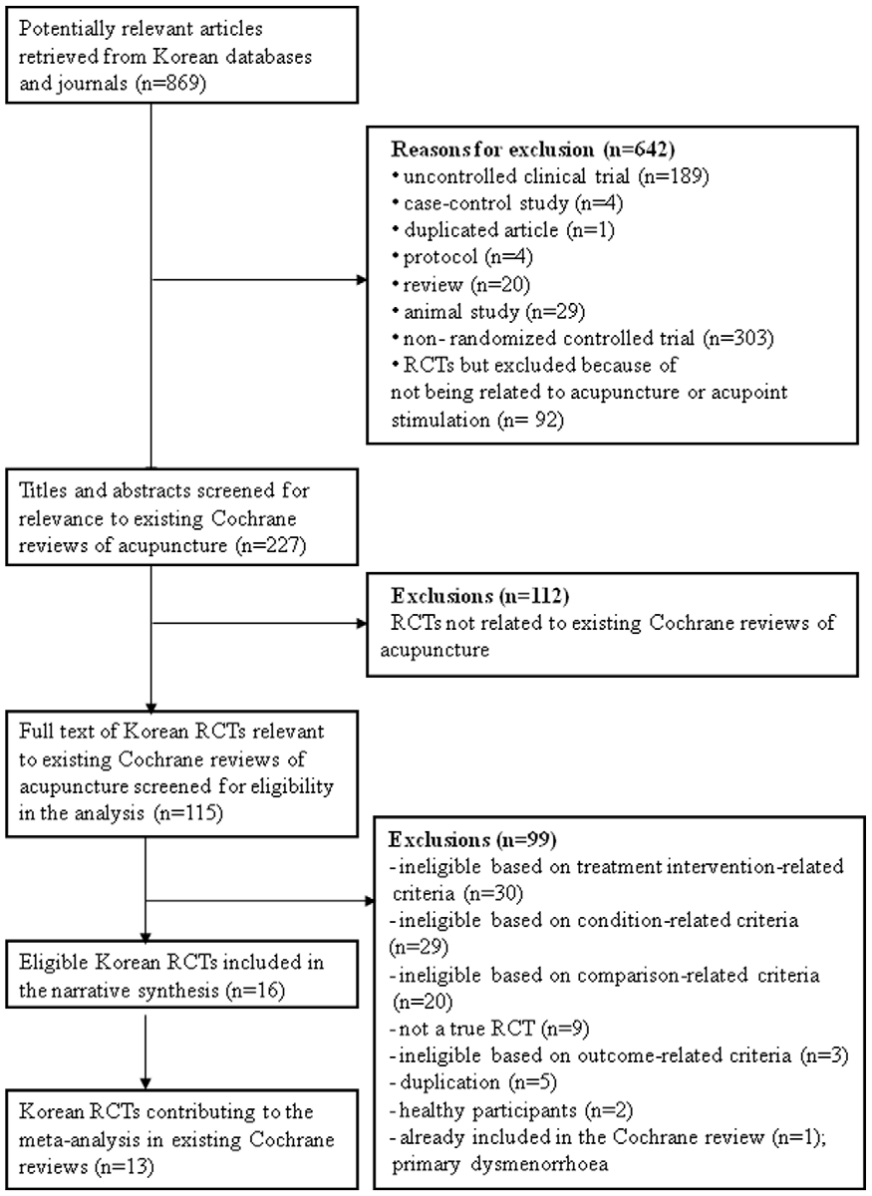
Flowchart of trial selection process. RCT  =  randomized controlled trial.

**Figure 2:**
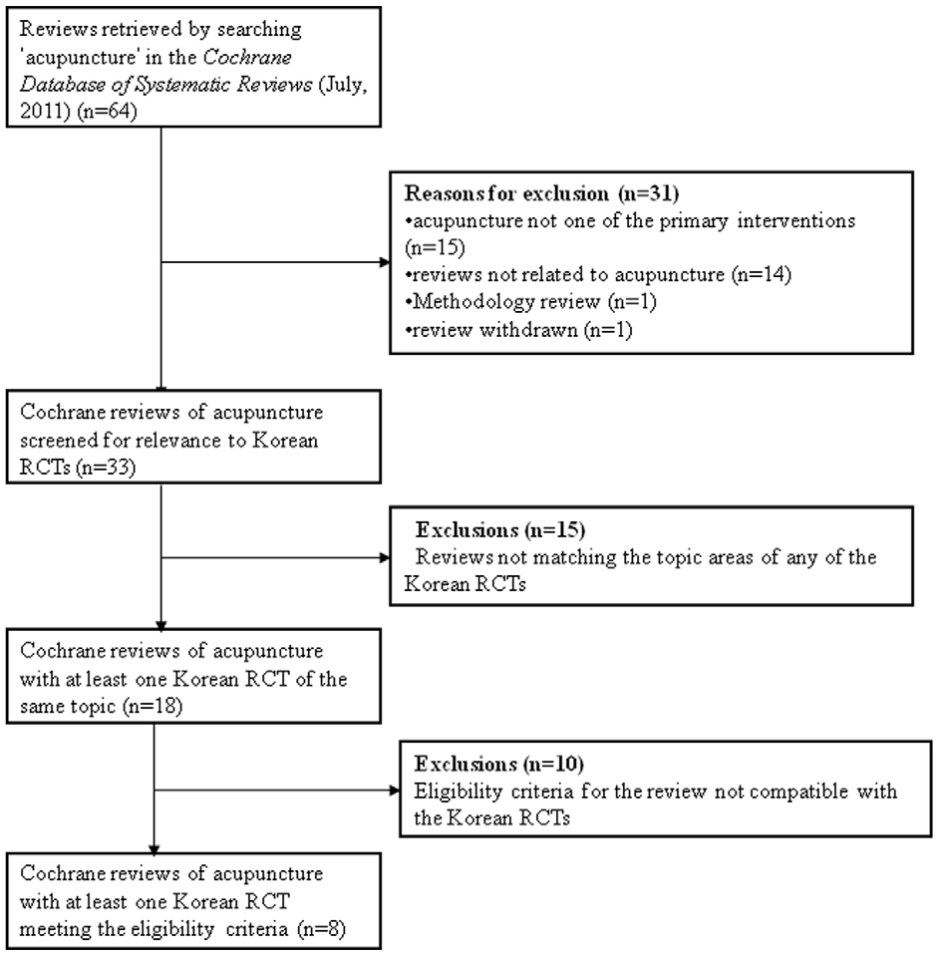
Flowchart of review selection process. RCT  =  randomized controlled trial.

**Table 2:** Screening results for potentially eligible Korean RCTs for relevant Cochrane reviews.

Cochrane review topics	No language restriction	Number of Korean Studies		
		At screening	Excluded	Eligible
Included (n = 8)				
Low back pain	Yes	21	16	5
Shoulder pain	Yes	13	10	3
Preventing postoperative nausea and vomiting	Yes	4	2	2
Insomnia	Unclear	4	2	2
Tension-type headache	Unclear	3	2	1
Primary dysmenorrhoea	Yes	6	5	1
Neck disorders	Yes	9	8	1
Cancer pain in adults	Yes	1	0	1
Excluded (n = 10)				
Lateral elbow pain	Yes	3	3	0
Stroke rehabilitation	Unclear	27	27	0
Dysphagia in acute stroke*	No	1	1	0
Induction of labor	Yes	1	1	0
Smoking cessation	Unclear	1	1	0
Pain management in labor	Yes	1	1	0
Rheumatoid arthritis**	No	1	1	0
Chemotherapy-induced nausea and vomiting	Yes	1	1	0
Peripheral OA	Yes	17	17	0
Bell's palsy	Yes	12	12	0

* : Studies reported in English and Chinese were searched.

** : Studies reported in English and French were searched.

While the importance of including Chinese databases in Cochrane systematic reviews of acupuncture is highlighted, the potential influence upon Cochrane or non-Cochrane acupuncture reviews of controlled trials in East Asian databases, other than those in Chinese, remains largely unknown. In our pilot study using the January 2011 issue of CDSR, 59 Cochrane reviews and protocols that regarded acupuncture as a primary intervention were identified [10]. The number of Cochrane reviews or protocols that included at least one Chinese database in their search was significantly higher (44 out of 59) than those in the study of Lui et al., [9] whereas the number that included at least one Korean database search was much smaller (4 out of 59). Although the number of Cochrane acupuncture reviews and protocols that include Chinese databases in the search is increasing, the lack of relevant database searches using languages other than English or Chinese still increases the susceptibility of these searches to the risk of language bias. Currently, there is no information on the influence of Korean papers reported in the Korean language upon Cochrane acupuncture reviews. To minimize potential language bias and ensure that comprehensive searchesare used to update Cochrane acupuncture reviews in future, the value of additional searching of Korean databases needs to be tested and its potential influence explored.

### Study objective

This study aimed to investigate whether the inclusion of searches of Korean databases might alter the results of Cochrane acupuncture reviews. We were also interested in any additional information that might be brought out by the hypothetical inclusion of Korean databases in the existing Cochrane acupuncture reviews.

## Methods

### Eligibility criteria

Randomized controlled trials (RCTs) written in Korean or English and indexed in Korean databases were eligible for this study. We decided not to include Korean RCTs written in English and indexed in English databases, because these studies might already have been identified as potentially eligible RCTs and thus could not serve the purpose of this study.

RCTs of patients with any particular health problems or diseases that corresponded to existing Cochrane acupuncture reviews were eligible. RCTs of healthy individuals were excluded.

Parallel group or crossover RCTs that involved any type of acupuncture point stimulation as treatment interventions, such as needle acupuncture, acupressure, device-involved acupuncture point stimulation (i.e., wrist band application) were deemed eligible. RCTs that employed moxibustion (a heat stimulation on acupuncture points using herbal preparations containing *Artemisia vulgaris*) [11] as a primary treatment intervention were excluded because we defined minimum criteria of acupuncture to involve a mechanical stimulation of predefined points (i.e., meridian points, Ashi points or local trigger points). RCTs providing *pharmacopuncture* (i.e., herbal injectionon acupuncture points) as a treatment intervention were only included if the Cochrane review clearly mentioned the inclusion of these studies or included these studies in the results. Otherwise, those studies were excluded.

### Searching methods and study-review selection process

For the initial selection of relevant Cochrane acupuncture reviews, the July 2011 issue of CDSR was searched using the term “acupuncture.” Reviews that considered acupuncture as a primary intervention were eligible for this study. Protocols and reviews that included acupuncture as one of various interventions were excluded. The topics of Cochrane reviews were screened and selected to identify whether the condition in the review related to those in the Korean RCTs. The texts of relevant Cochrane reviews were read in full for further analysis.

As for identifying the Korean RCTs that were able to be matched with the topics of published Cochrane acupuncture reviews, one author (JCK) conducted searches of controlled clinical trialsof acupuncture in 12 Korean academic portal databases (i.e., NANET, RISS4U, KISS, DBpia, KMbase, KoreaMed, KISTI, NDSL, OASIS, Dlibrary, KoreanTK, and RICHIS) from the time of their inception to July 2011. Unpublished theses and dissertations were also searched. In most Korean electronic databases, only simple Boolean searches were available. As not all Korean journals related to acupuncture are registered in the Korean academic portal databases, studies recorded electronically on the websites of seven acupuncture-related journals were also searched to ensure completeness of the search process. Since some Cochrane reviews included quasi-randomized trials, and because our experience showed that Korean RCTs did not always clearly demonstrate in the titles or abstracts that they were randomized, we included studies whose methods of randomization seemed doubtful at an initial screening phase. A list of search terms and journals searched is provided in Table 1.

For the initial selection of Korean RCTs, one author (JCK) examined the titles and abstracts obtained from the initial search and selected all potentially relevant studies. At this stage, only explicitly unrelated studies, including animal studies, surveys, narrative reviews, and case reports that could be identified by titles and abstracts, were excluded.

Titles and abstracts of screened studies were examined to check whether the topics corresponded to those of existing Cochrane reviews. Korean RCTs that did not match the topics of existing Cochrane reviews were discarded. The same author performed all the aforementioned study-selection processes.

The full texts of selected studies were then reviewed independently by three pairs of reviewers (KHK-JYC, BCS-JCK, and MSL-TYC) matched according to their clinical specializations. The purpose of this step was to select RCTs that met the specific eligibility criteria for each Cochrane review and to conduct the assessment of the risk of bias. The entire data extraction, excluding assessment of the risk of bias, was conducted by two authors independently. No attempt was made to conceal the names of the authors, institutions, or journals that published the original studies. We attempted to resolve any disagreements among the authors by convening monthly whole-group discussions over a period of six months, producing as great a degree of consensus as possible.

### Data extraction

Data extraction of eligible Korean RCTs was performed as follows: general trial information (year of publication, sample size); assessment of the risk of bias using the same criteria describedin the corresponding Cochrane review; trial outcomes as defined by the relevant Cochrane review. For reviews of low back pain trials, the Cochrane Back Group criteria were used to assess methodological quality [12]. Otherwise, risk of bias was assessed according to the Cochrane Handbook [13]. General information about trials already included in the relevant Cochrane acupuncture reviews were also extracted and compared to those of eligible Korean RCTs, to determine whether there were any trends showing differences in trial characteristics. The frequency of each database searched in Cochrane acupuncture reviews was counted to produce a descriptive summary.

### Comparison between Korean RCTs and studies included in the Cochrane reviews in regard to methodological quality

Similar to previous research , assessment of the risk of bias was conducted for two domains (i.e., allocation concealment and assessor blinding) in order to enable comparison between Korean RCTs and studies included in the Cochrane reviews with respect to their methodological quality [7]. Previous research has found that inadequately performed allocation concealment and assessor blinding significantly overestimate the effects of study interventions [14], [15]. The number of trials showing low versus high or unclear risk of bias in the two domains was compared.

### Data pooling and sensitivity analysis

To identify whether newly included Korean RCTs influenced the previous results of the Cochrane acupuncture reviews, sensitivity analyses were performed for augmented and new meta-analyses. The original RevMan files for each Cochrane review were downloaded from *The Cochrane Library* (www.thecochranelibrary.com). We defined a meta-analysis as the effect estimation of pairwise comparison for a certain outcome using statistical pooling of at least two sets of study data, regardless of whether total or subtotal estimation was calculated [2]. Augmented meta-analyses were defined as those with at least two alreadyexisting studiesplus at least one Korean study. New meta-analyses were defined as those having at least two studies after the inclusion of the Korean studies. The number of augmented meta-analyses, of new meta-analyses and of forest plots with a single Korean study after the inclusion of studies from Korean databases was recorded.

Differences in the size and direction of effect estimates before and after the inclusion of the Korean RCTs were investigated. For augmented meta-analyses, changes of statistical heterogeneity presented by I^2^ scores were also compared. I^2^ scores of 25 percent, 50 percent and 75 percent were regarded as corresponding to low, moderate, and high levels of heterogeneity. Any change in the heterogeneity level in the augmented meta-analyses (e.g., from low to moderate) was considered as a significant change of heterogeneity. Where available, a sensitivity analysis was also performed to identify whether the inclusion of the Korean RCTs altered funnel plot asymmetry of the meta-analysis. For effect size estimation, the decision to use a fixed-effect or a random-effects model was made according to the methods in each Cochrane review. When this was not clearly mentioned in the relevant Cochrane review, the random-effects model was preferred, taking account of the possible clinical heterogeneity that may have been attributable to the inclusion of Korean RCTs. The standardized mean difference (SMD) was used for continuous outcomes and risk ratio (RR) for dichotomous outcomes, respectively.

### Statistical analysis

Statistical analyses were performed using the SAS statistical package, version 9.1.3 (SAS Inc., Cary, NC, USA), and a two-sided p-value less than 0.05 was regarded as the level of statistical significance. Differences of general characteristics between trials in the Cochrane reviews and those in the Korean RCTs were tested using the chi-square test for dichotomous variables and t tests for continuous variables. Sensitivity analysis was performed using the RevMan software, version 5.1 (The Nordic Cochrane Centre, Copenhagen, Denmark).

## Results

A total of 869 articles were identified by the initial search of the Korean databases. (Additional searches of seven Korean acupuncture-related journals did not yield any new articles.) Of these, 642 clearly ineligible articles were excluded after screening the titles and abstracts. From among the remaining 227 potential studies, trials that did not match with topics of published Cochrane acupuncture reviews were further excluded (n = 112). The remaining 115 potentially relevant trials from Korean databases were examined in full, to investigate whether they met the eligibility criteria of the 18 topic-relevant Cochrane acupuncture reviews; this yielded a total of 16 studies (742 participants) that were eligible in eight Cochrane reviews (125 trials; 13,041 participants) (See the flowchart Figure 1 and 2, Table 2). Brief reasons for exclusion of Korean RCTs at this stage are provided in supporting information (Table S2).

The search term “acupuncture” in the *Cochrane Database of Systematic Reviews* (Issue 7, July 2011) yielded 64 reviews. Of these, 31 reviews were excluded for the following reasons: acupuncture was one of the treatment interventions but not the primary intervention (n = 15); reviews were not related to acupuncture (n = 14); methodology review (n = 1); review withdrawn (n = 1).

The relevance of the remaining 33 Cochrane reviews to the diseases or conditions investigated in the Korean RCTs was examined. Fifteen Cochrane acupuncture reviews did not match any Korean RCTs at the screening phase. Topics of excluded reviews included: chronic asthma, breech presentations, schizophrenia, acute strokes, cocaine dependence, irritable bowel syndrome, glaucoma, vascular dementia, restless leg syndrome, migraine prophylaxis, depression, uterine fibrosis, epilepsy, traumatic brain injury and attention deficit hyperactivity disorders. As a result, 18 Cochrane reviews that had at least one Korean study with the same topic were identified. Further investigation revealed that 10 reviews did not have any eligible Korean RCTs and were thus excluded from the analysis, leaving eight Cochrane reviews for this analysis.

The 16 hypothetically eligible Korean RCTs usedneedle acupuncture (n = 12), acupressure (n = 2), and transcutaneous electrical acupoint stimulation (n = 2) as treatment interventions. Diseases or conditions covered by Korean RCTs included low back pain (n = 5), shoulder pain (n = 3), insomnia (n = 2), prevention of postoperative nausea and vomiting (n = 2), tension-type headaches (n = 1), primary dysmenorrhea (n = 1), cancer pain in adults (n = 1), and neck disorders (n = 1). Eleven (69%) of the 16 Korean RCTs were published in acupuncture or traditional Korean medicine (TKM) journals (Table 3).

**Table 3:** Characteristics of RCTs already included in the Cochrane reviews and the hypothetically eligible Korean RCTs.

	Trials in the Cochrane reviews (n = 125)	Korean studies (n = 16)
Total number of participants in trials	13,041	742
Mean (SD)	105 (134)	46(19)
Median (Range)	68 (10–1265)	48 (12–86)
Number of studies in different conditions*		
Low back pain	35 (2861)	5 (226)
Neck disorders	10 (661)	3 (43)
Shoulder pain	9 (525)	3 (205)
Tension type headache	11 (2317)	1 (32)
Primary dysmenorrhea	10 (1025)	1 (47)
PONV	40 (4858)	2 (126)
Insomnia	7 (590)	2 (52)
Cancer pain in adults	3 (204)	1 (11)
Journal fields		
Acupuncture/TKM	-	11
Nursing/Physiotherapy	-	2
Conventional medicine	-	2
PhD thesis	-	1

RCTs: randomized controlled trials.

SD: standard deviation.

PONV: postoperative nausea and vomiting.

TKM: traditional Korean medicine.

* Values are provided as number of trials and (total number of participants).

Summary characteristics of the 16 Korean RCTs are provided in supporting information (Table S1) and should contribute to future updates of the eight Cochrane reviews being investigated in this study. Compared to the component studies included in the Cochrane acupuncture reviews, the risk of bias in the Korean trials was higher in terms of outcome assessor blinding and allocation concealment although the difference did not reach statistical significance in terms of allocation concealment (Table 4).

**Table 4:** Methodological quality of trials included in the eight topic-matched Cochrane reviews and the corresponding16 Korean studies.

	Trials in the Cochrane reviews (n = 125)	Korean studies (n = 16)	P*
Adequate concealment of allocation			0.1192
Yes	34 (27.2%)	1 (6.2%)	
No/Unclear	91 (72.8%)	15 (93.8%)	
Outcome assessor blinding			0.0136
Yes	75 (60%)	4 (25%)	
No/Unclear	50 (40%)	12 (75%)	

* Fisher's exact test.

Values are presented as number (%).

Databases searched in the eight Cochrane acupuncture reviews are illustrated in Table 5. The most frequently searched databases were MEDLINE, EMBASE and CENTRAL. The number of reviews that included searches of Chinese and Japanese databases was four and one respectively. One review searched both Chinese and Japanese databases. None of the eight reviews documented attempts to search Korean databases.

**Table 5:** Frequency of each database searched in the eight topic-matched Cochrane acupuncture reviews.

	Number of reviews, n (%)
English databases	
MEDLINE	8 (100.0)
EMBASE	7 (87.5)
CENTRAL	7 (87.5)
Cochrane Review Group specialized register	3 (37.5)
CINAHL	3 (37.5)
AMED	3 (37.5)
PsycInfo	2 (25.0)
Specialist acupuncture database	1 (12.5)
Other databases	7 (87.5)
East Asian databases	
Chinese databases	4 (50.0)
Japanese databases	1 (12.5)
Korean databases	0 (0.0)

Six of the eight Cochrane acupuncture reviews reported that no language restriction had been imposed (Table 2). Two reviews did not mention whether language restrictions had occurred, [16], [17] although one of these included Chinese database searching [16]. The language of publication of trials already included in the eight Cochrane acupuncture reviews consisted of English (n = 102), Chinese (n = 12), Japanese (n = 7), German (n = 2), Norwegian (n = 1), and Polish (n = 1).

Among 120 meta-analyses in the eight Cochrane reviews, the inclusion of the 16 Korean RCTs contributed to 24 existing meta-analyses, seven new meta-analyses and 50 new forest plots containing a single Korean RCT (Table 6). Inclusion of the Korean trials did not change the direction of effect in any of the existing meta-analyses. In the 24 meta-analyses augmented by the inclusion of Korean RCTs, the effect estimates became more beneficial in 13 meta-analyses, less beneficial in fourand did not change in the remaining six. For the outcome of side effects in the Cochrane low back pain review, no effect estimates were possible, although the result of the relevant Korean trial was combined into existing forest plot. Twelve out of the 13 meta-analyses in which the effect estimate became more beneficial showed a change in favor of the intervention of less than four percentage points. The remaining one meta-analysis showed 207% beneficial effects towards treatment interventions compared to no treatment in the reviews of insomnia [16]. However, heterogeneity significantly increased (from zero to 95%). In the four meta-analyses which became less beneficial after the inclusion of Korean RCTs, the percentage change of effect estimates ranged between 1% and 25%. In only one meta-analysis did the significance change (RR 0.78, [0.59, 1.02] I^2^ = 37% to RR 0.76 [0.59, 0.98] I^2^ = 37%) in one Cochrane review [18].

**Table 6:** Changes of meta-analysis after the inclusion of 16 Korean RCTs in relevant eight topic-matched Cochrane reviews.

	Numbers
Meta-analyses before inclusion of Korean RCTs	120
Meta-analyses augmented by Korean RCTs	24
New Meta-analyses after the inclusion of Korean RCTs	7
Forest-plots newly generated with a single Korean RCT	50
Meta-analyses augmented by Korean RCTs	24
Direction of effect changed	0
Effect size changed	1
Significance changed	1
Heterogeneity changed	2

Three out of the 7 new meta-analyses (of low back pain) showed significant effect estimates in favor of the treatment interventions. All of newly generated meta-analyses showed less than 15% heterogeneity.

Five of the eight Cochrane reviews had an additional 50 single-study forest plots after the inclusion of Korean RCTs. Among these, seven forestplots showed significant between-group differences. 90% (45 out of 50) of single-study forest plots belonged to the review of shoulder pain [19].

There was only one Cochrane acupuncture review that had a meta-analysis with at least 10 studies after the inclusion of Korean RCTs [18]. However, inclusion of the Korean RCTs did not change the funnel plot asymmetry.

Effect estimates and 95% confidence intervals of meta-analyses and single-study forest plots generated after the inclusion of Korean trials are provided in supporting information (Table S3, S4 and S5).

## Discussion

To the best of our knowledge, this is the first study that has evaluated the impact of including studies in individual languages on the existing results of Cochrane reviews. Hypothetical eligibility testing of the Korean literature using the same eligibility criteria as that used for selected Cochrane acupuncture reviews found a noticeable number of Korean RCTs that could have been included in the Cochrane reviews, had Korean databases been searched as part of the review process. In most cases, the inclusion of Korean RCTs did not change the result of the meta-analyses. Korean RCTs identified from the Korean databases added, at most, two studies with a small number of participants, in any of the individual forest plots. Nevertheless, a considerable number of new analyses became available by inclusion of Korean RCTs, suggesting that new information could be gained by inclusion of Korean databases in the search methods of Cochrane acupuncture reviews.

Risk of bias in the 16 hypothetically eligible Korean RCTs was higher in the domain of outcome assessor blinding and allocation concealment. Whether trials in languages other than English (LOE) that have a higher risk of bias should be included in evidence synthesis or not remains controversial. This is because studies with high risk of bias could be associated with exaggerated effect estimates [20]. However, previous empirical studies have suggested that there is no evidence of significant differences in terms of methodological quality between trials in English and those in LOE [21], [22]. Systematic searches for eligible studies regardless of the language of publication are recommended since a core component of systematic reviews is to ensure their validity and comprehensiveness [8], [23]. Given the controversy, one reasonable option would be to perform sensitivity analysis based on the trial quality and publication language. None of the Cochrane acupuncture reviews considered in our study conducted a sensitivity analysis according to publication language (i.e., trials reported in English versus LOE trials). Only one review attempted to perform a sensitivity analysis based on the publication country, but it failed to do so because of the paucity of component studies [16]. Four out of eight reviews attempted to perform sensitivity analysis based on the quality of trials, [16], [19], [24], [25] but only one was successful, again due to the paucity of component studies in the other three reviews [16], [24], [25]. From the viewpoint of Korean trialists, more attention should be devoted to maintaining methodological rigor, minimizing risk of bias, and adhering to the high quality of trial reporting guidelines (i.e., CONSORT) to maximize the potential benefit of including Korean RCTs in systematic reviews. Editors of Korean domestic journals should guide trial authors toward fulfilling all of the relevant reporting items of CONSORT in order to improve the reporting quality. Empirical evidence indicates that published Korean RCTs in conventional medicine show low adherence to the CONSORT guidelines [26]. Low adherence to these guidelines was also found in a traditional Chinese medical journal [27]. Collaborative efforts among Korean researchers and journal editors to improve methodological and reporting quality may bring about the inclusion of Korean RCTs in future Cochrane reviews of acupuncture.

Inclusion of Korean RCTs seemed unlikely to contribute sufficiently to the number of included trials for sensitivity analysis in any of the included Cochrane reviews. In future Cochrane reviews of acupuncture, as well as those analyzing healthcare interventions which have been performed in various cultural contexts and countries, sensitivity analyses of the inclusion of trials in English may be a reasonable option to assess whether language-restrictive analyses makes a difference to the robustness of review results compared to language-inclusive ones, as well as to secure both the comprehensiveness of the trial search process and the reliability of the evidence. No language restriction was declared in six of the eight Cochrane reviews. However, the language of publication included in those Cochrane reviews was mostly English. Our findings correspond to those of a previous study showing that only half of the 159 meta-analyses that reported language-inclusive searches had located studies published in languages other than English [7]. Possible reasons might be poor participation of Korean authors in the conduct of Cochrane acupuncture reviews. In our pilot study, only four Korean authors were found to have participated in Cochrane acupuncture reviews in the January 2011 issue of CDSR [10]. Low awareness among international researchers about research activity, as well as the clinical practice of acupuncture in Korea, might also have played a role in the omission of Korean literature from current evidence syntheses. Appropriate training and education for enhancing the participation of Korean researchers in Cochrane acupuncture reviews will contribute to increasing the inclusion of Korean literature in future evidence syntheses.

Methods to improve accessibility of Korean literature for international researchers should also be investigated. We are aware that controlled clinical trials of acupuncture in the Korean literature are being registered in the Cochrane Central Register of Controlled Trials (CENTRAL) by Korean researchers and the Cochrane CAM Field. This will accelerate the identification of Korean RCTs and the testing of their eligibility, thus reducing the potential risk of language bias and maximizing completeness of current and future evidence of acupuncture available in CDSR. Collaborative efforts for incorporating local evidence into Cochrane reviews, such as those being made by the Chinese Cochrane Center and CONSORT groups to improve the quality of reporting in RCTs published in Chinese languages, [28] are needed between Korean and international researchers to overcome the incompleteness of the search strategies addressed in this study.

A relatively large number of Korean RCTs did not satisfy the eligibility criteria of existing Cochrane acupuncture reviews and were excluded from the analysis. A substantial number of Korean RCTs employed different comparisons, different styles of acupuncture, and different outcomes from those in the Cochrane reviews, hence they were ultimately excluded from the reviews. This might be partly due to the existence of research questions and priorities among Korean acupuncture researchers that are different from those of Cochrane acupuncture reviewers, although in the context of Korea, no information is available for the research priorities relating to acupuncture. A recent survey showed that practice characteristics and research priorities of practitioners of traditional acupuncture were different in China and Europe [29]. Acupuncture is a complex intervention, in which the whole process of patient consultation and therapeutic interaction comprises overall effectiveness, and these are significantly influenced by cultural and societal backgrounds [30], [31]. Competing local priorities for research and research interests of those involved in trials might be different in different countries. This might partly explain the high rate of exclusion from the Cochrane reviews of Korean studies, due to ineligible comparison (i.e., comparing two different acupuncture techniques). Future research focusing on factors that potentially determine the research questions and trial designs in the field of acupuncture research in the Korean situation might be helpful in explaining gaps between evidence generated in Korea and evidence generated by Cochrane systematic reviews, reflecting current variability in the field of acupuncture research and practice.

### Differences between the findings of this study and previous research

Previous research has assessed whether language-inclusive meta-analyses make a difference to the results compared to language-restrictive ones, by conducting sensitivity analyses for trials reported in languages other than English [7], [23]. In one study, only meta-analyses which had included trials reported in both English and LOE were collected [7]. One major weakness of this approach is that review authors assume that meta-analyses being analyzed had included all relevant LOE trials by comprehensive and adequate search methods, which was clearly not the case in our findings. Although our study included only a limited number of Cochrane reviews and Korean RCTs in terms of a specific intervention (i.e., acupuncture), it showed that even Cochrane reviews with language-inclusive searches had omitted a certain proportion of eligible LOE RCTs. This means that the impact of LOE trials might have been underestimated in previous research studies that only included given study sets in meta-analyses [7]. Based on our findings, we suggest that Cochrane reviews of acupuncture should pay more attention to develop adequate methods to access and identify LOE trials. Future research that evaluates the impact of language bias should also consider the risk of omission of LOE trials when using already included study sets in meta-analyses, unless searches of hypothetically eligible studies could be performed by the researchers themselves.

### Strengths of this study

First, to the best of our knowledge, the largest number of Korean databases and relevant acupuncture journals published in Korean were searched for this study. Future Cochrane acupuncture reviews and protocols might refer to this study for developing search strategies that include Korean literature. Second, study summaries and information on excluded studies are available in supporting information (Table S1 and S2) for existing and future Cochrane review authors, thus making this study more informative for concerned researchers. A summary of 16 hypothetically eligible Korean RCTs found in this study is provided and could be directly integrated into forthcoming updates of existing Cochrane acupuncture reviews. Third, we have tried to develop the data set of acupuncture RCTs published in Korean literature, which could be periodically updated and used as an important source of building regional data sets for Korean acupuncture RCTs. We also suggest future collaborative activity for incorporating evidence of acupuncture in Korean literature covered by this study into CENTRAL, one of the most important databases for systematic reviews.

### Limitations of this study

Limitations of this study should be discussed. First, search terms used in this study for locating controlled acupuncture trials in Korean literature might not be optimal. To the best of our knowledge, however, there is no standard search filter for the most efficient identification of RCTs in Korean databases. We attempted to overcome this weakness by extensively searching all relevant electronic databases and performing ancillary searches in related journals. To address this limitation, development of sensitivity-maximizing search filters for Korean RCTs is needed. Second, only a small number of Korean RCTs were eligible in this analysis. However, we tried to screen all relevant studies in several Korean databases by methods stated above; thus, we believe we have used the most representative set of acupuncture trials reported in Korean databases. Third, initial searching and screening for relevant Korean studies was performed by only one assessor since the process was labor-intensive and research resources did not extend to independent screening. Instead, three pairs of two independent researchers at the post-screening stage selected eligible RCTs, assessed the risk of bias, and extracted data. Fourth, only published Cochrane reviews were screened for this analysis. Current ongoing protocols and registered review titles that might be relevant to Korean RCTs were not considered in the analysis. Thus, the results of this study might be outdated when reviews are completed and updated in the near future. Continuous efforts, regional and international research activities, and research funding are needed to maintain an up-to-date body of evidence for the practice of acupuncture in Korea and for the incorporation of such evidence into CDSR. Lastly, only Cochrane reviews that considered acupuncture as a primary treatment intervention were included in this study. Hence, the impact of inclusion of Korean RCTs on existing Cochrane reviewsfor other health-related fields might not be fully assessed.

### Conclusions

Inclusion of Korean databases in the search methods for Cochrane systematic reviews of acupuncture can add valuable information to enhance current evidence of the use of acupuncture in relevant clinical fields. Inclusion of Korean RCTs should be considered for any Cochrane reviews in preparation and for future revisions and periodic updates of existing Cochrane acupuncture reviews.

## Supporting Information

Table S1 **Summary characteristics and risk of bias of included 16 Korean studies.**
(DOC)

Table S2 **Characteristics of 99 excluded Korean studies.**
(DOC)

Table S3 **Seven new meta-analyses after the inclusion of Korean trials.**
(DOC)

Table S4 **24 augmented meta-analyses after the inclusion of Korean trials.**
(DOC)

Table S5 **50 Single-study forest plots generated by the inclusion of Korean studies.**
(DOC)
