# Supplementary material for: Impact of Including Korean Randomized Controlled Trials in Cochrane Reviews of Acupuncture
Source: PLoS One. 2012 Oct 11;7(10):e47619. doi: 10.1371/journal.pone.0047619 (PMC3469498; doi:10.1371/journal.pone.0047619)
Supplement: Table S1 — Summary characteristics and risk of bias of included 16 Korean studies. (DOC) [file pone.0047619.s001.doc]

Table S1. Summary characteristics and risk of bias of included 16 Korean studies

1. Relevant review: Acupuncture and dry-needling for low back pain

| Study information | Study ID: Kwon 2007 [1]  Title: The short-term efficacy of acupuncture for chronic low back pain: randomized sham controlled trial.  Reference details: Journal of Oriental Rehabilitation Medicine2007;17(2):123-132.  Correspondance: Kwon, Young-Dal. E-mail: [kwondy@wonkwang.ac.kr](mailto:kwondy@wonkwang.ac.kr) |
| --- | --- |
| Methods | - Randomized: a computerized randomization program. Allocation concealment unclear (not reported)  - Patient and assessor blinded  - Funding: Korea Institute of Oriental Medicine  - Setting: Won-kwang University hospital. Gwang-ju, South Korea. Subjects were recruited via hospital homepages and posters  - Informed consents and ethics approval: written consent obtained. Ethic approval obtained.  - All patients were analyzed by ITT principle although 3 dropouts were reported,  - Analysis: repeated measure ANOVA, student's t-test and chi-square test were used.  All values were described as Mean ± standard deviation.  , sham-controlled, parallel clinical trial, two groups: 1) manual acupuncture 2) sham acupuncture (non-acupoint, superficial (1-2mm) insertion without any stimulation and attempts to elicit de-qi response)  Assessment of blinding:  Patient: Questions to test blinding effectiveness were asked at the end of the treatment  Assessor: Outcome measures were assessed by the 'blinded' assessor  Groups comparable at baseline: no differences between two groups  Lost to follow up: 57 subjects were screened and 50 (87.7%) were enrolled…. Of the 50 subjects who enrolled in the study, 25 subjects were randomized to the acupuncture group and 25 were randomized to the sham group…………… Three patients (MA n=1, SA n=2) dropped out of the study. Reasons for dropping out included living too far away to comply with the treatment schedule (n=2), and no contact (n=1). These dropouts were included for ITT analysis.  Compliance: not reported  Duration: June, 2006 to July, 2006.  Intention-to-treat analysis: done. |
| Participants | 50 patients  Male/Female: 17/30 (from patients who have completed the trial)  Age: Not reported  Inclusion criteria: 1) Lumbar or lumbosacral pain for duration of three months or longer; patient over 20 years old 2) LBP must be the chief complaint 3) Having normal neurological examinations 4) Having an IRB-approved consent form in his/her own hand.  Exclusion criteria: 1) Potential lumbar disease(e.g. a spinal tumor, infection or lumbar fracture; etc); 2) other diseases (e.g. bleeding disease, dementia, epilepsy, neurogenic disorder or systemic disease etc.) 3) Planned lumbar surgery 4) Prior use of acupuncture within the past 6 months 5) Current use of systemic corticosteroids, narcotics, anticoagulants, or muscle relaxants 6) Involvement in any legal problem related to LBP 7) Refusal to be randomized  How low-back pain associated with potential lumbar disease was excluded: All patients had a plain X-ray and CT of L-spine on their initial visit  Diagnosis: Chronic Lumbar or Lumbosacral pain  Working status: not reported  Previous Treatments: Not reported  Co-morbidity: Not reported |
| Interventions | 25 patients randomized in each group  Subjects in the acupuncture group were treated with three sessions of manual acupuncture weekly for 4 weeks; i.e. 12 sessions in total. Manual stimulation was performed by ‘reinforcing and reducing by lifting and thrusting the needle' once in 20 minutes, and subjects felt *De-qi* sensations. Disposable stainless needles (0.25mmx40mm, Dong-bang, Korea) were inserted into the acupoints to a depth of 25-30mm. Points were chosen for individual patients as in routine clinical practice based on the traditional Korean medicine classic (*Dong Ui Bo Gam),* using points on the back, hand, and legs. Acupoints used were SI3, BL62, BL60, BL40, CV4, BL23, BL52, BL29, CV2 and GB30.  Control group: Subjects in the sham group were treated with three sessions of minimal acupuncture weekly for 4·weeks; i.e. 12 sessions in total. Manual stimulation was not used during the 20-minute period and subjects didn't feel *De-qi* sensations. Disposable stainless needles (0.25mmx40mm, Dong-bang, Korea) were inserted into non-acupoints superficially, 10~20mmaway from the acupoints of MA group described previously to a depth of 1-2mm with 38-39mm of needle length with guide tube.  Each treatment was conducted by the same practitioner (a traditional Medical Doctor registered by the Korean government) |
| Outcomes | Primary outcome measures  1) Pain intensity, quantified using a -100 mm Visual Analogue Scale (VAS)  2) Pain disability, measured using Roland Disability Questionnaire *(RDQ).* The RDQ consists of 24 questions to be answered yes or no (range 0-24 points, most severe=24).  The VAS and RDQ scores were recorded at 0 week, 2 weeks and 4 weeks.  Secondary outcomes  1) Patient Global Assessment (PGA  2) Digital Thermography (DT).  The PGA was recorded at intervals of 0 week, 2 weeks, and 4 weeks. Digital Thermography (Digital Infrared Thermographic Imaging: DITl, DorexInc, Orange, CA, USA) was assessed on GB21, BL23, GB30, BL57 at both 0 week and 4 weeks in a room with a temperature maintained at 23-24 'C, and humidity of 60-70%.  Questions to test blinding effectiveness were asked at the end of treatment, and any side effects were recorded after respective treatment.  Outcome measures were assessed by the 'blinded' assessor.  Costs: not reported  Complications: Fatigue. Treatment Group: N=1 (prior experience with acupuncture, after 2nd treatment) Control Group: N= 3 (2 with prior acupuncture experience, 1 with no experience, all occurred after 1st treatment). No serious adverse events reported. |
| Notes | Language: English  Publication: Full paper  Additional information from authors: not sought  Outcomes (VAS, RDQ, patient global scale) were converted as change scores from baseline to be included in the meta-analyses.  Conclusion: Manual acupuncture was effective for chronic LBP, but not necessarily superior to the sham results. (from Discussion) |

**Assessment of Risk of Bias: Kwon 2007 [1]**

| **Domain** | **Low risk of bias?**  Yes/No/Unclear* | | | **Description**  **(With Reasons of rating risk of bias)** |
| --- | --- | --- | --- | --- |
| **A. Was the allocation sequence adequately generated?** |  |  |  | They were randomly sorted into two groups using a computerized randomization program. |
| **B. Was allocation adequately concealed?** |  |  |  | Not reported (Selected through a 3^rd^ party) |
| **C. Were the groups similar at baseline regarding the most important prognostic indicators?** |  |  |  | Similar to each group. See Table 1 in the original studies (age, body weight, gender, location of pain, onset of pain, symptom duration of pain were not statistically different between each group) |
| **D. Was the patient blinded to the intervention?** |  |  |  | More than 70% of participants in each group guessed that they received real acupuncture treatment. |
| **E. Was the care provider blinded to the intervention?** |  |  |  | Each treatment was conducted by the same practitioner |
| **F. Was the outcome assessor blinded to the intervention?** |  |  |  | Outcome measures were assessed by the blinded assessor. |
| **G. Were cointerventions avoided or similar?** |  |  |  | No cointerventions reported |
| **H. Was the compliance acceptable in all groups?** |  |  |  | Three patients (MA n=1 in treatment group, SA n=2 in control group) dropped out of the study. |
| **I. Was the drop-out rate described and acceptable?** |  |  |  | Three patients (MA n=1, SA n=2) dropped out of the study. Reasons for dropping out included living too far away to comply with the treatment schedule (n=2), and no contact (n=1). These drop-outs were included for ITT analysis. |
| **J. Was the timing of the outcome assessment in all groups similar?** |  |  |  | Two and four weeks from baseline in each group |
| **K. Did the analysis include an intention-to-treat analysis?** |  |  |  | These drop-outs were included for ITT analysis. |

* Note: For each section above ‘Yes’ indicates a ‘low risk of bias’; ‘No’ indicates a ‘high risk of bias’; ‘Unclear’ indicates an ‘uncertain risk of bias’.

| Study information | Study ID: Lee 2011 [2]  Title: The comparison of effectiveness between acupuncture and its cotreatment with Wan-Gwa acupuncture on the treatment of low back pain  Reference details: Journal of Korean Acupuncture and Moxibustion Society. 2011;28(2):43-47.  Correspondance: Song, Beom-yong. E-mail: [acudoctor@korea.com](mailto:kwondy@wonkwang.ac.kr) |
| --- | --- |
| Methods | Random methods: Outpatients were randomized into two groups. No details on random methods were provided. No details on allocation concealment.  Outcome assessors: no details on assessor blinding reported  Funding: Woosuk university  Setting: Outpatient clinic in South Korea.  Informed consent: Not reported  IRB: Not reported  Follow-up: 27 of 42 randomized participants  Analysis: No ITT. Mann-Whitney U-tests for between group differences and Wilcoxon signed rank test for within group differences |
| Participants | Inclusion criteria: 1) lumbar sprain diagnosed by traditional Korean medicine (TKM) doctor 2) age from 20 to 70 3) symptom duration less than 1 month 4) No lumbar disease found by the plain lumbar radiography at baseline assessment  Exclusion criteria: 1) previous history of spinal surgery 2) vertebral compression fracture found by the radiography 3) sustained lumbar radiculopathy 4) currently other treatments 5) congenital spinal disease  How low-back pain associated with potential lumbar disease was excluded: only patients with no abnormality by the plain lumbar radiography were eligible.  Diagnosis: Low back  Working status: not reported  Previous Treatments: Not reported  Co-morbidity: Not reported  Male/Female: 7/22 (from patients who have completed the trial)  Age[years]: 44.55 ±4.77 (in Treatment group); 43.30 ±10.11 (in Control group) |
| Interventions | Three sessions of manual acupuncture weekly for 2 weeks, i.e. 6 sessions in total. Both group received the same acupuncture treatment except the needling of additional points on Ha6.  Sterilized disposable stainless needles were used  Needle length and diameter: 0.30 X 40 mm, DongBang acupuncture. Co. Korea  Needling depth: 20-30mm according to patient’s condition  Stimulation: Manual stimulation. Plain tonification and reduction technique. No information whether De-qi sensation were elicited or not.  Needle retention time: 20 minutes in total  Needling points: BL23, BL24, BL25, BL26, BL27 (bilaterally) and GB29, GB30, GB34, BL40 (unilaterally on affected side)  In Treatment group: Additional points on Ha6 (located laterally on Achilles tendon), superficial insertion, 20 minutes retention  Cupping: BL23, BL24, BL25 BL26 unilaterally on affected side (8 minutes)  Physical treatment: Interferential current therapy; ultrasound; hot pack on low back region.  Each treatment was conducted by the same practitioner (TKM doctor) |
| Outcomes | 1. Pain: VAS (0 to 10; 10 as the most severe pain, 0 as a pain-free condition) 2. K-ODI (9 items, no sexual life-related question)   Pain VAS was taken at baseline, mid-treatment (twice a week), post-treatment. However, only baseline and post-treatment values were reported.  No information on frequency of K-ODI evaluation was provided in Methods. K-ODI results at baseline and post-treatment were reported in Results.  Costs: not reported  Complications: unclear (not reported for the occurrence of adverse event) |
| Notes | Language: Korean  Publication: Full paper.  Additional information from authors: not sought  Conclusions: ”Both body plus ankle acupuncture and body acupuncture alone have significantly reduced pain VAS and ODI scores compared to pre-treatment conditions in each group. Body plus ankle acupuncture have significantly reduced ODI scores compared to body acupuncture alone” |

**Assessment of Risk of Bias: Lee 2011 [2]**

| **Domain** | **Low risk of bias?**  Yes/No/Unclear* | | | **Description**  **(With Reasons of rating risk of bias)** |
| --- | --- | --- | --- | --- |
| **A. Was the allocation sequence adequately generated?** |  |  |  | Not reported about the method of random sequence generation |
| **B. Was allocation adequately concealed?** |  |  |  | Not reported |
| **C. Were the groups similar at baseline regarding the most important prognostic indicators?** |  |  |  | No statistically significant differences in terms of age, gender and baseline pain and function level between two groups |
| **D. Was the patient blinded to the intervention?** |  |  |  | Comparing between two different acupuncture techniques |
| **E. Was the care provider blinded to the intervention?** |  |  |  | Acupuncture practitioner could not be blinded in this study. |
| **F. Was the outcome assessor blinded to the intervention?** |  |  |  | Not reported |
| **G. Were cointerventions avoided or similar?** |  |  |  | Both groups received the same treatment except the Ha6 acupuncture. |
| **H. Was the compliance acceptable in all groups?** |  |  |  | 13 of 42 patients dropped-out. (31% drop-out rate) |
| **I. Was the drop-out rate described and acceptable?** |  |  |  | 13 of 42 patients dropped-out. (31% drop-out rate)  No information on drop-out in each group were reported. No reasons for drop-out were provided. |
| **J. Was the timing of the outcome assessment in all groups similar?** |  |  |  | All patients were assessed after the two-week treatment course. |
| **K. Did the analysis include an intention-to-treat analysis?** |  |  |  | Analysis were based on the 29 participants (15 for additional Ha6 acupuncture group and 14 for standard acupuncture group). 13 of 42 patients dropped-out. (31% drop-out rate) |

| Study information | Study ID: Kim 2010 [3]  Title: A clinical study on effect of electro-acupuncture treatment for lumbago patients caused by traffic accident  Reference details: Journal of Korean Acupuncture and Moxibustion society. 2011;27(5):117-123.  Correspondance: Yoo, In-sik. E-mail: 12theo@naver.com |
| --- | --- |
| Methods | Random methods: Outpatients were randomized into two groups. No details on random methods were provided. No details on allocation concealment.  Outcome assessors: no details on assessor blinding reported  Funding: Not reported  Setting: Outpatient clinic in the specialized hospital for spinal disorders in South Korea.  Informed consent: Not reported  IRB: Not reported  Follow-up: 60 patients enrolled, no report on the attrition during the study period.  Analysis: Whether ITT analysis was conduction remains unclear due to lack of description. Independent samples t-test for between-group comparison of the mean in the VAS and RMDQ scores. Paired samples t-test for within group differences. |
| Participants | Inclusion criteria: 1) pain and discomfort on lumbar region after traffic accident  Exclusion criteria: 1) patients with cognitive disorder not enough to cooperate the study instruction 2) current use of pacemaker 3) patients who did not agree to receive electoacupuncture  How low-back pain associated with potential lumbar disease was excluded: only patients with simple lumbar sprain confirmed by lumbar radiography were included.  Diagnosis: lumbar sprain  Working status: not reported  Previous Treatments: Not reported  Co-morbidity: Not reported  Male/Female: 33/27  Age[years]: 36.40 ±12.77 (in Treatment group); 37.20 ±11.73 (in Control group) |
| Interventions | For both groups: prone position. Stainless steel needle acupuncture 0.30 * 40 mm (DongBang Acupuncture, Korea). Needling depth 10-20 mm. Needle retention time 15 min. Electroacupuncture device was STN-111, Stratec. Acupuncture points were: GV4, GV3, BL23, BL24, BL25, BL26, A-shi points on the low back region.  Twice a week for 4 weeks, totally 8 sessions of acupuncture treatments were provided in both groups.  1) Group 1 (Acupuncture with electrostimulation): After the investigator performed needling, leads were connected. Another TKM doctor who does not know the study purpose turned on the electrostimulator and gradually increased the intensity of electrical stimulation.  2) Group 2 (Acupuncture with pretended mock electrostimulation): After the investigator performed needling, leads were connected. Another TKM doctor who does not know the study purpose turned on the electrostimulator, but only beep sounds were provided without any real electrical stimulation.  Concomitant treatment: Infrared radiation on the needled region. Herbal extracts (O-Juk San; 五積散) were provided in both groups. |
| Outcomes | 1. Pain: VAS (0 to 10; 0 as a pain-free condition and 10 as an undurable severe pain) 2. RMDQ (24 items)   Pain VAS were at baseline, 1-, 2-, 3-, 4-week from baseline (after 2-, 4-, 6-, 8-session, respectively)  RMDQ were measured at baseline, 2- and 4-week from baseline (after 4-and 8-session, respectively).  Costs: not reported  Complications: unclear (not reported for the occurrence of adverse event) |
| Notes | Language: Korean  Publication: Full paper.  Additional information from authors: not sought  Conclusions: “There was no significant difference between the two groups in the VAS and RMDQ in the statistics. However, it turned out that electro-acupuncture and general acupuncture was effective in reducing the pain of the patients in group A (real electrostimulation) and B (mock electrostimulation) according to increased number of the treatment.” |

**Assessment of Risk of Bias: Kim 2010[3]**

| **Domain** | **Low risk of bias?**  Yes/No/Unclear* | | | **Description**  **(With Reasons of rating risk of bias)** |
| --- | --- | --- | --- | --- |
| **A. Was the allocation sequence adequately generated?** |  |  |  | No detail for the randomization method provided. |
| **B. Was allocation adequately concealed?** |  |  |  | Unclear. Not reported. |
| **C. Were the groups similar at baseline regarding the most important prognostic indicators?** |  |  |  | No significant between-group difference in terms of age, pain and function disability level at baseline |
| **D. Was the patient blinded to the intervention?** |  |  |  | Authors reported that they attempted to blind both patients and investigators by involving the separate practitioner after the leads were connected. |
| **E. Was the care provider blinded to the intervention?** |  |  |  | Authors reported that they attempted to blind both patients and investigators by involving the separate practitioner after the leads were connected. |
| **F. Was the outcome assessor blinded to the intervention?** |  |  |  | No details on assessor blinding reported. Pain VAS was self-administered by the patient. However, who collected the outcome remains unclear. |
| **G. Were cointerventions avoided or similar?** |  |  |  | Concomitant treatment: Infrared radiation on the needled region. Herbal extracts (O-Juk San; 五積散) were provided in both groups. |
| **H. Was the compliance acceptable in all groups?** |  |  |  | Not reported |
| **I. Was the drop-out rate described and acceptable?** |  |  |  | Not reported |
| **J. Was the timing of the outcome assessment in all groups similar?** |  |  |  | Similar in both groups. |
| **K. Did the analysis include an intention-to-treat analysis?** |  |  |  | Not reported |

| Study information | Study ID: Lee 2009 [4]  Title: The comparative study on effect of body acupuncture and 8 constitution acupuncture in acute stage lumbago-for patients checked up the Pancreotonia-  Reference details: Journal of Korean Acupuncture and Moxibustion society. 2009;26(2):181-188.  Correspondance: Yoo, In-sik. E-mail: 12theo@naver.com |
| --- | --- |
| Methods | Random methods: Outpatients were randomized into two groups. According to the sequence of visiting number, patients with odd number were allocated to the Group 1 (8-constitutional acupuncture) and those with even number to the Group 2 (Body acupuncture) Not possible to perform the allocation concealment.  Outcome assessors: no details on assessor blinding reported  Funding: Not reported  Setting: Outpatient clinic in the TKM hospital in Pohang, South Korea.  Informed consent: Not reported  IRB: Not reported  Follow-up: 50 patients enrolled. Although no report on the attrition during the study period, it is unlikely that attrition had occurred because outcomes were measured before and after a single treatment session.  Analysis: Whether ITT analysis was conducted remains unclear due to lack of description. Within-group differences measured by the Wilcoxon signed rank test, Between-group comparison by the Mann-Whitney U test and Chi-Square test. |
| Participants | Inclusion criteria: 1) patients with the constitution of the Pancreotonia (土陽) in terms of 8-constitutional acupuncture theory 2) Acute low back pain within 6-week of symptom occurrence.  Exclusion criteria: not reported  How low-back pain associated with potential lumbar disease was excluded: not reported  Diagnosis: acute low back pain  Working status: not reported  Previous Treatments: Not reported  Co-morbidity: Not reported  Male/Female: 19/31  Age[years]: 39.16 ±9.99 (in Group 1); 37.36 ±10.22 (in Group 2)  Symptom duration [days]: 5.12 ± 5.35 (in Group 1); 4.4 ± 4.16 (in Group 2) |
| Interventions | 1) Group 1 (8-constitutional acupuncture): Supine position. Unilateral insertion (Right side according the the acupuncture theory). A special needle for 8-constitutional acupuncture (Hanglim medical device, Korea) (other specifications not reported) were used. Needling depth 3-5mm. Repetitive rapid insertion and immediate withdrawal of needle per each point. (Acupuncture formula for spinal disorder comprised of basic, internal organ and spiritual formula; basic formula comprised of 5-times manipulation of SP3, KI3, LU8, KI7 5; internal organ formula comprised of 5-times manipulation of HT8, SP2, KI10, SP9; spiritual formula comprised of one-time manipulation of HT7, HT3)  2) Group 2 (Acupuncture with pretended mock electrostimulation):  Lateral position. Stainless steel needle acupuncture 0.25 * 30 mm (Hanglim medical device, Korea). Needling depth 10-20 mm. Needle retention time 15 min.  Acupuncture points were: BL23, BL24, BL25, BL26, GV3. Additional points were allowed according to the patients’ symptom. (BL31, BL32, BL33, BL34, GB30).  Concomitant treatment: No concomitant treatments were allowed (non-study acupuncture, herbal medicine, physical therapy, cupping therapy).  Practitioner information: not reported |
| Outcomes | 1. Pain: VAS (10 to 0; 10 as baseline pain level) 2. Range of motion (Lumbar flexion, extension)   Pain VAS and ROM were measured at pre-treatment assessment (baseline) and immediate after treatment (post-treatment)  Costs: not reported  Complications: unclear (not reported for the occurrence of adverse event) |
| Notes | Language: Korean  Publication: Full paper.  Additional information from authors: not sought  Conclusions: “8 constitution acupuncture therapy can be recommended as a useful therapy to treat patients checked up the Pancreotonia with acute stage lumbago. Further study is needed to confirm the effectiveness of 8 constitution acupuncture.” |

**Assessment of Risk of Bias: Lee 2009 [4]**

| **Domain** | **Low risk of bias?**  Yes/No/Unclear* | | | **Description**  **(With Reasons of rating risk of bias)** |
| --- | --- | --- | --- | --- |
| **A. Was the allocation sequence adequately generated?** |  |  |  | Randomization based on the sequence of visiting number |
| **B. Was allocation adequately concealed?** |  |  |  | Not reported |
| **C. Were the groups similar at baseline regarding the most important prognostic indicators?** |  |  |  | Baseline pain level was not reported as it is. (standardized as 10 points on the VAS) |
| **D. Was the patient blinded to the intervention?** |  |  |  | Not possible |
| **E. Was the care provider blinded to the intervention?** |  |  |  | Not possible |
| **F. Was the outcome assessor blinded to the intervention?** |  |  |  | not reported. |
| **G. Were cointerventions avoided or similar?** |  |  |  | No concomitant treatments were allowed (non-study acupuncture, herbal medicine, physical therapy, cupping therapy). |
| **H. Was the compliance acceptable in all groups?** |  |  |  | Presumably no attrition because of a single session treatment and before-after assessment. |
| **I. Was the drop-out rate described and acceptable?** |  |  |  | Presumably no attrition because of a single session treatment and before-after assessment. |
| **J. Was the timing of the outcome assessment in all groups similar?** |  |  |  | Yes. Similar in all groups. |
| **K. Did the analysis include an intention-to-treat analysis?** |  |  |  | Whether ITT analysis was conducted remains unclear due to lack of description. However, presumably no attrition because of a single session treatment and before-after assessment. |

| Study information | Study ID: Min 2002 [5]  Title: The effects of the needle electrode electrical simulation in the auricular therapy on the smokers and non-smokers with low back pain  Reference details: Journal of Korean Physical Therapist [translated] 2002;9(1):45-52.  Correspondance: Park, Su-jin. E-mail: not reported |
| --- | --- |
| Methods | - Randomized: methods of randomization not reported. Allocation concealment unclear (not reported)  - Patient and assessor blind: not reported  - Outcome assessors: no details on assessor blinding reported  - Funding: not reported  - Setting: physical therapist clinic, South Korea. Subjects were recruited from outpatients presented in the clinic.  - Informed consents and ethics approval: both not reported.  - Follow-up: 24 patients enrolled. Not reported about the attrition rates.  - Analysis: Whether ITT analysis was conducted remains unclear due to lack of description. Paired t-test for comparison of the changes of mean. |
| Participants | Inclusion criteria: 1) patients with more than 3-month symptom duration of ischemic chronic low back pain and without other musculoskeletal or endocrinal diseases 2) (For smoking group) Daily smoking more than 15 cigarettes and less than 2 hours after smoking when presenting the clinic. 3) (For non-smoking group)Nosmoking from at least 3 years ago.  Exclusion criteria: not reported  How low-back pain associated with potential lumbar disease was excluded: not reported  Diagnosis: chronic ischemic low back pain  Working status: not reported  Previous Treatments: Not reported  Co-morbidity: Not reported  Male/Female: Not reported  Age[years]: 49.50 ±4.03 (in Group 1); 51.50 ±3.78 (in Group 2); 44.33 ± 7.42 (in Group 3); 49.50 ± 5.99 (in Group 4) |
| Interventions | 1) Group 1 (n=6): Needle electrode electrical stimulation (NEES) and conventional physical therapies for smoking patients  2) Group 2 (n=6): Conventional physical therapies for smoking patients  3) Group 3 (n=6): Needle electrode electrical stimulation and conventional physical therapies for non-smoking patients  4) Group 4 (n=6): Conventional physical therapies for smoking patients  Treatment sessions: daily treatment for 5 days in each group.  For NEES; Disposable piriform needles (Hansol medical device, Korea) were used (no other needle specification reported). Needling depth 2.0 mm. Auricular points included the Occiput, Lung, Internal Nose, Shenmen, Phalynx-Larynx, Endocrine. Electrostimulation (Device: WQ-6F[China]; 3 Hz frequency with the intensity just below the pain threshold, 20 minutes)  For conventional physical therapies; transcutaneous electrical nerve stimulation (CS-210; Japan) (3 Hz frequency with the intensity just below the visible muscle contraction, 20 minutes), Ultrasound therapy (SUS-2N; Korea)  Concomitant treatment: Not reported  Practitioner information: not reported |
| Outcomes | 1. Pain: VAS (0 to 10; the meaning of ranges not reported) 2. Pain: Verbal rating scale (5-point): No pain, mild, moderate, severe, very severe pain 3. Pain: McGill pain questionnaire 4. Serum beta-endorphin   Costs: not reported  Complications: unclear (not reported for the occurrence of adverse event) |
| Notes | Language: Korean  Publication: Full paper.  Additional information from authors: not sought  Conclusions: “Based upon the results, it can be concluded that smokers with low back pain received the more effects of pain alleviation from the application of the needle electrode electrical stimulation in the auricular therapy compared to the rest of the groups who suffered from low back pain.”  Reviewer comment: For reasons of meaningful analysis, four groups were merged into two groups (Auricular acupuncture group plus conventional physical therapy versus conventional physical therapy alone) when data were entered into the Revman. |

**Assessment of Risk of Bias: Min 2002 [5]**

| **Domain** | **Low risk of bias?**  Yes/No/Unclear* | | | **Description**  **(With Reasons of rating risk of bias)** |
| --- | --- | --- | --- | --- |
| **A. Was the allocation sequence adequately generated?** |  |  |  | Not reported |
| **B. Was allocation adequately concealed?** |  |  |  | Not reported |
| **C. Were the groups similar at baseline regarding the most important prognostic indicators?** |  |  |  | Yes. Age, baseline pain level. |
| **D. Was the patient blinded to the intervention?** |  |  |  | Not possible |
| **E. Was the care provider blinded to the intervention?** |  |  |  | Not possible |
| **F. Was the outcome assessor blinded to the intervention?** |  |  |  | Not reported. |
| **G. Were cointerventions avoided or similar?** |  |  |  | The same conventional physiotherapies were applied in each group. |
| **H. Was the compliance acceptable in all groups?** |  |  |  | Not reported |
| **I. Was the drop-out rate described and acceptable?** |  |  |  | Not reported |
| **J. Was the timing of the outcome assessment in all groups similar?** |  |  |  | Yes. Similar in all groups. |
| **K. Did the analysis include an intention-to-treat analysis?** |  |  |  | Not reported |

2. Relevant review title: Acupuncture for shoulder pain

| Study information | Study ID: Nam 2007b [6]  Title: Clinical observation of acupuncture and nerve block treatment for adhesive capsulitis patients  Reference details: Journal of Korean Acupuncture and Moxibustion Society 2007;24(4):159-171  Correspondance: Choi Do-Young. E-mail :choi4532@unitel.co.kr |
| --- | --- |
| Methods | - Random allocation: Yes. Random Table.  - Concealed allocation: unclear. Not reported  - Baseline comparability: Yes; no statistically significant differences between three groups in terms of age, gender, symptom duration, mode of onset and baseline outcome scores.  - Blind assessors: Unclear; not reported  - Blind participants: Not possible; open trial  - Blind therapists: Not possible; open trial  - Adequate follow-up: one-month from baseline  - Intention-to-treat analysis: No. only patients who completed the study were included in the analysis.  - Between group comparisons: Yes  - Point estimates and variability: Yes; mean and standard deviation.  - Eligibility criteria: Yes  - Funding: Supported by KRF (Korea Research Foundation) and Kyung Hee University, 2005  - Setting: Kyung Hee University hospital, Seoul, South Korea. Subjects were recruited via newspaper, community broadcasting and hospital homepages.  - Informed consents and ethics approval: written consent obtained. Ethic approval obtained.  Lost to follow up: 59 subjects were enrolled. Eight of 59 patients were dropped-out. Reasons include suggestive cancer (n=1), participant withdrawal due to unsatisfactory allocation results (n=3), personal reason (n=1) and too long distance to participate in the trial (n=3). These drop-outs were not included in the analysis.  Compliance: not reported  Duration: May, 2006 to September, 2006. |
| Participants | 59 patients enrolled, 51 patients analyzed.  Male/Female: 24/35  Age: 56.37 ±6.84  Inclusion criteria: 1) chronic shoulder pain with symptom duration of one to 12 months 2) significant limitation on the range of motion for passive and active movement 3) nocturnal shoulder pain and inability to lie laterally  Major Exclusion criteria: 1) Previous history of major shoulder injuries or shoulder surgery 2) Other pathological conditions that may explain the shoulder pain 3) Cervical myelopathy or neurological defects that may be associated with shoulder pain 4) fracture, inflammatory arthritis, liver or kidney disease, hematopoietic disorders, malignant cancer, psychiatric disease 5) ROM limitation between 40 to 120 degree abduction with suggestive diagnosis of rotator cuff injuries |
| Interventions | Group 1 (n=22); manual acupuncture on two non-classical points (Gyeon Jung, Shin gwan points) and local points (LI15, TE14, GB21) twice a week for four weeks. 0.30 * 40 mm disposable stainless needles (Dongbang acupuncture, ChungNam) were used. Manual stimulation with 15 minutes of needle retention time.  Group 2 (n=17): Nerve block techniques twice a week for four-weeks. Suprascapular nerve block using 5-8ml of 1% lidocaine with steroids injected on the suprascapular fossa. Subacromial injection with 5 ml of 1% lidocaine with steroids, and trigger point injection using 0.5 to 2 ml of local anaesthesia injection.  Group 3 (n=20): Acupuncture combined nerve block therapy (treatments as the same in the group 1 and 2).  Self-exercise recommended for all groups  Information on practitioners for acupuncture or nerve block therapy: not reported |
| Outcomes | 1. Constant Shoulder Assessment  2. Shoulder Pain and Disability Index  3. Visual Analogue Scale  4. Range of motion (shoulder flexion, extension, abduction and adduction)  5. Skin temperature on both shoulder region by Digital Infrared Thermography  Assessed at baseline, 1-, 2-, 3-, 4-week from baseline (for outcome 1 to 4)  Assessed at baseline and 4-week from baseline (for outcome 5)  Complications: unclear (not reported for the occurrence of adverse event) |
| Notes | Language: Korean  Publication: Full paper  Additional information from authors: not sought  Conclusion: Acupuncture and nerve block combination treatment for adhesive capsulitis patients is more effective than the two single treatments. |

**Assessment of Risk of Bias: Nam 2006b [6]**

| **Domain** | **Low risk of bias?**  Yes/No/Unclear* | | | **Description**  **(With Reasons of rating risk of bias)** |
| --- | --- | --- | --- | --- |
| **Was the allocation sequence adequately generated?** |  |  |  | Using random table |
| **Was allocation adequately concealed?** |  |  |  | Not reported. |
| **Blinding: Participant** |  |  |  | Not possible |
| **Attempt to confirm patient blinding for sham control** |  |  |  | Not applicable |
| **Blinding: Assessor** |  |  |  | Not reported |
| **Blinding: Other participants** |  |  |  | Not reported |
| **Incomplete outcome report** |  |  |  | 86 % of enrolled participants completed the trial and included in the analysis. |
| **Selective outcome reporting** |  |  |  | Protocol information not accessible. |
| **Other sources of bias** |  |  |  | Non-remarkable |
| **Notes** |  |  |  |  |

| Study information | Study ID: Nam 2007a [7]  Title: Randomized controlled trial of East-West collaborate medical treatment on female chronic shoulder pain patients  Reference details: Journal of Korean Acupuncture and Moxibustion Society 2007;24(6):113-122  Correspondance: Choi Do-Young. E-mail: choi4532@unitel.co.kr |
| --- | --- |
| Methods | - Random allocation: Yes. Randomization code.  - Concealed allocation: unclear. Not reported  - Baseline comparability: Yes; no statistically significant differences between three groups in terms of age, symptom duration and baseline outcome scores.  - Blind assessors: Yes; authors reported that single blind was successfully applied by a separated outcome assessor.  - Blind participants: Not possible; open trial  - Blind therapists: Not possible; open trial  - Adequate follow-up: one-month from baseline  - Intention-to-treat analysis: No. only patients who completed the study were included in the analysis.  - Between group comparisons: Yes  - Point estimates and variability: Yes; mean and standard deviation.  - Eligibility criteria: Yes  - Funding: Supported by KRF (Korea Research Foundation) and Kyung Hee University, 2005  - Setting: Kyung Hee University hospital, Seoul, South Korea. Subjects were recruited from the call center agents working at the customer service center of the Korean gas cooperation.  - Informed consents and ethics approval: written consent obtained. Ethic approval obtained.  Lost to follow up: 60 subjects were enrolled. Six of 60 patients were dropped-out (two per each treatment groups). Reasons include failure to receive treatment due to working schedules (n=2), failed to show up at the final assessment (n=2), fear of needle injection (n=1), receiving another treatment without discussion (n=1). These drop-outs were not included in the analysis.  Compliance: not reported  Duration: April, 2007 to June, 2007 |
| Participants | 60female patients enrolled, 54 patients analyzed.  Age: 40.28 ±6.31 (Group 1), 37.89±6.96 (Group 2), 38.39±5.96 (Group 3)  Inclusion criteria: 1) shoulder pain for at least one month 2) appreciable pain of active or passive motion 3) Pain at night with inability to lie on the affected side  Exclusion criteria: 1) Previous history of major shoulder injuries or shoulder operation 2) Clinical evidence of other pathology that could possibly account for symptoms 3) Patients with cervical radiculopathy, paresis, or other neurological changes in the upper limb on the involved side 4) fracture, associated inflammatory arthritis, known renal or hepatic disease, hematopoietic disorders, malignant cancer, any mental disorder likely to interfere with the course of assessment of the disease process 5) Painful arc between 40 to 120 degree indicative of rotator cuff disease |
| Interventions | Group 1 (n=20); manual acupuncture on two non-classical points (Gyeon Jung, Shin gwan points) and local points (LI15, TE14, GB21) twice a week for four weeks. 0.30 * 40 mm disposable stainless needles (Dongbang acupuncture, ChungNam) were used. Manual stimulation with 15 minutes of needle retention time.  Nerve block techniques twice a week for four-weeks. Suprascapular nerve block using 5-8ml of 1% lidocaine with steroids injected on the suprascapular fossa. Subacromial injection with 5 ml of 1% lidocaine with steroids, and trigger point injection using 0.5 to 2 ml of local anaesthesia injection.  Group 2 (n=20): manual acupuncture on two non-classical points (Gyeon Jung, Shin gwan points) and local points (LI15, TE14, GB21) twice a week for four weeks. 0.30 * 40 mm disposable stainless needles (Dongbang acupuncture, ChungNam) were used. Manual stimulation with 15 minutes of needle retention time.  Group 3 (n=20): no treatment.  Self-exercise recommended for all groups.  Information on practitioners for acupuncture or nerve block therapy: not reported |
| Outcomes | 1. Constant Shoulder Assessment  2. Shoulder Pain and Disability Index  3. Visual Analogue Scale for subjective pain  Assessed at baseline and 4-week from baseline  Complications: unclear (not reported for the occurrence of adverse event) |
| Notes | Language: English  Publication: Full paper  Additional information from authors: not sought  Conclusion: Combination of acupuncture and nerve block injection may be an effective option in the treatment of chronic shoulder pain. |

**Assessment of Risk of Bias: Nam 2007a [7]**

| **Domain** | **Low risk of bias?**  Yes/No/Unclear* | | | **Description**  **(With Reasons of rating risk of bias)** |
| --- | --- | --- | --- | --- |
| **Was the allocation sequence adequately generated?** |  |  |  | Using randomization code |
| **Was allocation adequately concealed?** |  |  |  | Unclear. Not reported |
| **Blinding: Participant** |  |  |  | Not possible |
| **Attempt to confirm patient blinding for sham control** |  |  |  | Not applicable |
| **Blinding: Assessor** |  |  |  | authors reported that single blind was successfully applied by a separated outcome assessor. |
| **Blinding: Other participants** |  |  |  | Not applicable |
| **Incomplete outcome report** |  |  |  | 90 % of enrolled participants completed the trial and included in the analysis. |
| **Selective outcome reporting** |  |  |  | Protocol information not accessible. |
| **Other sources of bias** |  |  |  | Only female patients were included which might affect the subjective assessment of pain level. |
| **Notes** |  |  |  |  |

| Study information | Study ID: Lee 2006 [8]  Title: A clinical trial of acupuncture treatment for frozen shoulder  Reference details: Journal of Korean Acupuncture and Moxibustion Society 2006;23(1):165-177.  Correspondance: Lee Hyun.E-mail: [lh2000@hanmir.com](mailto:lh2000@hanmir.com) |
| --- | --- |
| Methods | - Random allocation: unclear. Not reported.  - Concealed allocation: unclear. Not reported.  - Baseline comparability: Yes; no significant differences between three groups in terms of age, gender, physical examination results and other patient characteristics.  - Blind assessors: unclear. Not reported.  - Blind participants: Yes; sham controlled trial.  - Blind therapists: Not possible.  - Adequate follow-up: one-month from baseline  - Intention-to-treat analysis: Yes.  - Between group comparisons: Yes  - Point estimates and variability: No; results provided as figures.  - Eligibility criteria: Yes  - Funding: Supported by the Korea Institute of Oriental Medicine, 2005.  - Setting: Dunsan University hospital, Daejeon, South Korea. Subjects were recruited via local newspaper, broadcasts, flyers, hospital homepages and postings.  - Informed consents and ethics approval: written consent obtained. Ethic approval obtained.  Lost to follow up: unclear. Not reported  Compliance: not reported  Duration: September, 2005 to December, 2005 |
| Participants | 86female patients enrolled, 86 patients analyzed according to the author report.  Age: 51.70 ±7.29 (Group 1), 51.39±6.00 (Group 2), 51.27±7.17 (Group 3)  Inclusion criteria: 1) Age between 40 and 65. 2) symptom duration between one and 6 months. 3) shoulder pain with the limitation of the range of motion. 4) clinically and radiographically diagnosed frozen shoulder.  Exclusion criteria: 1) radiographically confirmed calcification or shoulder arthritis with structural deformity 2) any mental or immune disorder 3) current use of corticosteroid treatment or non-steroidal anti-inflammatory drugs 4)cancer, psoriasis, neurological disorder, metabolic bone disease, acute injury or significant secondary deformative arthritis. 5) pregnant or breast-feeding 6) infectious skin disease |
| Interventions | The total number of participants (n=86) is not the same as the sum of three groups (n=85).  Acupuncture treatments three times weekly for 4 weeks. Totally 12 sessions. 30 minutes of needle retention time for all groups. No co-intervention or medication was allowed during the study period. Ipsilateral acupuncture points were selected with the manual stimulation of plain tonification and reduction techniques. 32-guage needles (Dongbang Acupuncture, Korea) were used.  Group 1 (n=28); Local points. Manual acupuncture on SI15, SI14, SI9, LU2, SI10, SI13, SI12, SI11 (8 points).  Group 2: semi-fixed local and distal points according to the pain area and diagnosis based on the meridian theory.  1) pain on the anterior region of shoulder (hand large intestine meridian): local points including LI15 and LI16 as well as distal points including LI4, LI11, LI10, LI14, ST38, ST36.  2) pain on the lateral region of shoulder (hand triple energizer meridian): local points including TE14 and TE15 as well as distal points including TE2, TE3, TE5, TE6, GB34, GB41.  3) pain on the posterior region of shoulder (hand small intestine meridian): local points including SI9 and SI10 as well as SI2, SI3, SI4, BL57, ST39, SI7.  Group 3: local non-meridian, non-acupuncture points (8 points. Located at random around the scapula)  Needling depth: varied according to the points based on the Acupuncture textbook (with reference) in Group 1 and 2. 1-2mm of minimal penetration in Group 3.  Information on practitioners for acupuncture treatments: not reported |
| Outcomes | Primary outcome:  1. pain VAS at motion  Secondary outcome:  1. Shoulder Pain and Disability Index  2. Patient global assessment (0 to 4 ordinal scale. 0: no pain; 1: slight pain; 2: moderate pain; 3: severe pain; 4: very severe pain)  3. shoulder ROM (abduction, adduction, lateral rotation, external rotation and internal rotation)  Assessed at baseline, 1-, 2- and 4-week from baseline.  Complications: unclear (not reported for the occurrence of adverse event) |
| Notes | Language: Korean  Publication: Full paper  Additional information from authors: not sought  Conclusion: Acupuncture at both the remote and the nearby acupoints according to the meridian theory is effective to improve external rotation of frozen shoulder, and acupuncture at the nearby acupoints is effective to improve adduction of frozen shoulder. However, it is not clear that acupuncture treatment at both the reome and the nearby acupoints according to the meridian theory is more effective than treating at only the nearby acupoints in the treatment of frozen shoulder. (Abstracts) |

**Assessment of Risk of Bias: Lee 2006 [8]**

| **Domain** | **Low risk of bias?**  Yes/No/Unclear* | | | **Description**  **(With Reasons of rating risk of bias)** |
| --- | --- | --- | --- | --- |
| **Was the allocation sequence adequately generated?** |  |  |  | Unclear. Methods not reported |
| **Was allocation adequately concealed?** |  |  |  | Unclear. Not reported |
| **Blinding: Participant** |  |  |  | Yes. Reported by authors. |
| **Attempt to confirm patient blinding for sham control** |  |  |  | Unclear. Not reported |
| **Blinding: Assessor** |  |  |  | Unclear. Not reported. |
| **Blinding: Other participants** |  |  |  | Not possible. |
| **Incomplete outcome report** |  |  |  | Authors reported that all included participants were analyzed. |
| **Selective outcome reporting** |  |  |  | Some of ROM changes were not reported. |
| **Other sources of bias** |  |  |  | Non-remarkable. |
| **Notes** |  |  |  |  |

3. Relevant review title: Acupuncture for dysmenorrhoea

| Study information | Study ID: Yoon 2008 (paper requiring translation in the review) [9]  Title: Effect of acupuncture treatment on the primary dysmenorrhea (a study of single blind, sham acupuncture, randomized, controlled clinical trial)  Reference details: Journal of Korean Acupuncture and Moxibustion Society 2007;25(3):139-156  Correspondance: Choi Sun Mi. E-mail :smchoi@kiom.re.kr |
| --- | --- |
| Methods | - Random allocation: Yes. Randomization table.  - Concealed allocation: unclear. Not reported  - Baseline comparability: Yes; authors reported that there were no statistically significant differences between two groups in terms of age, previous analgesic medication and symptom severity.  - Blind assessors: unclear; not reported  - Blind participants: Yes; authors reported that subjects could not know their allocation results.  - Blind therapists: Not possible; authors reported that the acupuncture practitioner was aware of allocation results.  - Intention-to-treat analysis: No. According to the authors’ report, 110 participants screened, 80 enrolled, 18 dropped-out, finally 25 in the treatment group and 22 in the control group were included in the analysis. The sum of analyzed patients did not match with the number of patients that subtracted 18 of dropped-out patients from 80 enrolled subjects. There may be more drop-outs during the study. Reasons of drop-out: absent from the required visits (n=10), found uterine fibroids and cysts during the study period (n=8).  - Compliance: not reported  - Duration: April, 2007 to June, 2007  - Funding: Supported by the Korea Institute of Oriental Medicine, 2007.  - Setting: Dong Eui University hospital, Pusan, South Korea. Subjects were recruited via newspaper advertisements and webpage postings.  - Informed consents and ethics approval: written consent obtained. Ethic approval obtained. |
| Participants | 80 female patients enrolled, 47 patients finished the trial and analyzed.  Inclusion criteria: 1) age between 18 and 40. 2) pain level scored more than 5 of 10 point scales. 3) significant disruption of daily function and social life due to dysmenorrhoea. 4) No underlying pathology which can account for dysmenorrhea diagnosed by the gynecologist specialist with abdominal ultrasound and blood test. 5) no history of abdominal surgery. 6) agreed to use no analgesic medication during the study period.  Exclusion criteria: 1) irregular menstrual periods (less than 20 days or more than 26 days) between previous three months. 2) history of any mental disorder. 3) systemic disease managed by medication during at least one year.4)history of hysterectomy. 5) current or planning pregnancy in 6 months. 6) current use of oral contraceptives. 7) secondary dysmenorrhoea |
| Interventions | Group 1 (n=25); Local and distal acupuncture points based on the theory of Traditional Korean medicine and Sa-am acupuncture theory. Points selected by literature reviews and previous research.  Points include : GB41, SI3, SI2, BL66, SP6, CV6. In case of Cold or deficiency syndrome, add ST36, and in case of Qi stagnation or excessive syndrome, add LR2. Needles were placed unilaterally (right side) and manipulated by clockwise-anticlockwise rotation with tonification and reduction techniques to elicit De-qi response.  0.40 * 50 mm acupuncture needle (DongBang Acupuncture, Korea) for CV6 in the depth of 30mm, and 2-3 times manual rotation with immediate withdrawal of the needle without retention. 20*30 mm acupuncture needle (DongBang Acupuncture, Korea) were used and retained 20 minutes for the rest of points. Needling depth for those points were 0.5 to 0.8 cun, determined by the Acupuncture textbook (Jipmoondang, Korea).  3 TKM doctors with 6-year university education at least 3-year clinical experience of acupuncture treatments were trained specifically for the study were involved in the administration of acupuncture treatments. These TKM doctors were instructed to maintain minimal interaction with participants.  Group 2 (n=22): sham acupuncture on non-acupuncture points (one cun away from TE4 and LU10, 0.5 cun away from CV8, ST40, GB40) (total 5 points). 0.20 *30 mm needles (Dongbang acupuncture) were inserted superficially (less than 3mm)and retained 20 minutes without any stimulation. De-qi response was not sought for sham acupuncture technique. Other needling details are the same as the treatment group.  Total treatment sessions: 8  Treatment frequency: once per 7 to 10 days.  Co-interventions: None. |
| Outcomes | 1.pain due to dysmenorrhea measured by the Measure of Menstrual Pain (MMP) scale (developed by the author).  2. pain VAS for average and maximum level, respectively.  3.symptom of dysmenorrheal measured by the Menstrual Symptom Severity List (MSSL) scale (developed by the author)  4. Patients’ global assessment (5-point likert scale; 1: no symptom; 2: markedly improved; 3: somewhat improved; 4: no change; 5: symptom deterioration)  Assessed at baseline, after 1^st^menstruation (after two sessions of acupuncture), after 2^nd^menstruation (after five sessions of acupuncture) and after 3^rd^ menstruation (after eight sessions of acupuncture).  Complications: Authors reported that they observed minor temporary irregularity of menstrual period (the number of case was not reported) as well as minor vaginal bleeding (n=1); however they were deemed irrelevant to acupuncture treatment by authors. |
| Notes | Language: Korean  Publication: Full paper  Additional information from authors: not sought  Conclusion: Acupuncture treatment was effective in decreasing the symptom of primary dysmenorrhea. |

**Assessment of Risk of Bias: Yoon 2008 [9]**

| **Domain** | **Low risk of bias?**  Yes/No/Unclear* | | | **Description**  **(With Reasons of rating risk of bias)** |
| --- | --- | --- | --- | --- |
| **Was the allocation sequence adequately generated?** |  |  |  | Yes. Random table used |
| **Was allocation adequately concealed?** |  |  |  | Unclear. Not reported |
| **Blinding: Participant** |  |  |  | Yes. Reported by authors. |
| **Attempt to confirm patient blinding for sham control** |  |  |  | Unclear. Not reported |
| **Blinding: Assessor** |  |  |  | Unclear. Not reported. |
| **Blinding: Other participants** |  |  |  | Not possible.Not possible; authors reported that the acupuncture practitioner was aware of allocation results. |
| **Incomplete outcome report** |  |  |  | 47 of 80 patients (59%) finished the study and were included in the analysis. |
| **Selective outcome reporting** |  |  |  | Study protocol was available from the authors. VAS was not clearly defined in the protocol, and reported as average/maximum pain during menstrual period, respectively. No clear description about the reporting method of variation (e.g., SD or SE) was available either in the protocol or in the publication. No SD or SE was reported in the publication. |
| **Other sources of bias** |  |  |  | The study reported baseline comparability only for those who have completed the study (n=47), but not for those who initially enrolled in the study (n=80). |
| **Notes** |  |  |  |  |

**4. Relevant review title: Acupuncture for insomnia**

| Study information | Study ID: Jung 2010 [10]  Title: Effect of auricular acupress therapy on insomnia of cancer patients: randomized, single blinded, placebo controlled trial  Reference details: Journal of Pharmacopuncture 2010;13(2):93-100.  Correspondance: Hwa-Seung Yoo. E-mail: altyhs@dju.kr |
| --- | --- |
| Methods | - Random allocation: Yes. Randomization table used.  - Concealed allocation: unclear. Not reported.  - Baseline comparability: Authors insisted that baseline characteristics were comparable in both groups, in terms of age, gender, type and stage of cancer.  - Blind assessors: unclear. Not reported.  - Blind participants: Yes; placebo controlled trial. (pressing auricular acupoints that are deemed irrelevant to the treatment of insomnia)  - Blind therapists: Not possible.  - Intention-to-treat analysis: No.  - Between group comparisons: Yes  - Funding: Supported by the Korea Institute of Oriental Medicine, 2005.  - Setting: East-West Cancer Center, Daejeon University hospital, Daejeon, South Korea. Subjects were recruited from inpatients of the university hospital.  - Informed consents and ethics approval: Not reported |
| Participants | 12 cancer patients enrolled, 7 patients finished the study.  Group 1: treatment group (males); 6 (3)  Group 2: control group (males); 6 (2)  Age: 52.8 ±10.6 (Group 1), 51.3±6.3 (Group 2)  Inclusion criteria: 1) Age between 20 and 75. 2) Less than 35 of Oh’s sleeping score. 3) No coexisting condition (pain or ascites) that could affect sleep status. 4) No mental disorder and ability to understand and comply with the treatment.  Exclusion criteria: 1) major physical or mental illness (stroke, psychiatric disease, dementia, major depressive disorder). 2) Inability or no willingness to discontinue sleep medication during the study period. 3) Patients wearing pacemaker or other transplanted electric device. 4) Infection or abscess on the external ear.  Associated disease: Cancer  Duration of disorder: Not reported  Previous treatments: Not reported |
| Interventions | Group 1 (Treatment): Auricular acupressure treatment on the ear Shenmen, insomnia, frontal region and neurasthenia point.  Group 2 (Control): Auricular acupressure treatment on the ear triple energizer point, shoulder and back point, clavicle point, dental extraction analgesia point. (deemed as irrelevant for the treatment of insomnia)  Non-penetrating studs (Angel Korea, Inc) attached. No details about stimulation methods of studs.  Duration of treatment: Studs were replaced at a 3-day interval. Totally 5 sessions were provided. (Total treatment periods were 15 days)  Information on practitioners for acupuncture treatments: not reported |
| Outcomes | Outcomes:  1. Oh’s sleeping score (ranged 15 to 60 scores; higher scores reflect better sleep).  2. Numeric rating scale (NRS) for insomnia  Table 3.  Oh’s sleeping score: Treatment group: 34.0 ± 4.3 (baseline), 39.5 ± 3.1 (post-treatment); Control group: 38.3 ± 3.5 (baseline), 40.0 ± 0.0 (post-treatment)  NRS: Treatment group: 6.3 ± 2.9 (baseline), 4.8 ± 2.1 (post-treatment); Control group: 7.0 ± 1.0 (baseline), 5.0 ± 2.6 (post-treatment)  Complications: unclear (not reported for the occurrence of adverse event) |
| Notes | Language: Korean  Publication: Full paper  Dropouts: 2 and 3 in treatment and control group, respectively. No reasons described.  Duration of follow-up: 15 days (from baseline)  Additional information from authors: not sought  Conclusion: Although both groups did not show significant differences, most of the experimental participants showed increasing OSS and NRS after treatments. Significant participants’ number will be needed in the next study. (Abstracts) |

**Assessment of Risk of Bias: Jung 2010 [10]**

| **Domain** | **Low risk of bias?**  Yes/No/Unclear* | | | **Description**  **(With Reasons of rating risk of bias)** |
| --- | --- | --- | --- | --- |
| **Was the allocation sequence adequately generated?** |  |  |  | Yes. Random table used |
| **Was allocation adequately concealed?** |  |  |  | Unclear. Not reported |
| **Blinding: Participant** |  |  |  | Yes. Reported by authors. |
| **Attempt to confirm patient blinding for sham control** |  |  |  | Unclear. Not reported |
| **Blinding: Assessor** |  |  |  | Unclear. Not reported. |
| **Blinding: Other participants** |  |  |  | Not possible. |
| **Incomplete outcome report** |  |  |  | 7 of 12 patients (58%) finished the study and were included in the analysis. |
| **Selective outcome reporting** |  |  |  | No study protocol was available. No clear description about the reporting method of variation (e.g., SD or SE) was available in the publication. |
| **Other sources of bias** |  |  |  | Other baseline characteristics (for example, duration of disorder or previous treatment history for insomnia) that might be associated with treatment effects were not reported) |
| **Notes** |  |  |  |  |

| Study information | Study ID: Sok 2005 [11]  Title: Effects of auricular acupuncture on insomnia in Korean Elderly  Reference details: J Korean Acad Nurs 2005;35(6):1014-1024.  Correspondance: Kim, Kwuy Byn. E-mail: kuikim@khu.ac.kr |
| --- | --- |
| Methods | - Random allocation: Unclear. Not reported about random allocation method.  - Concealed allocation: unclear. Not reported.  - Baseline comparability: Authors insisted that baseline characteristics were comparable in both groups, in terms of age, gender, education level, socioeconomic and marital status.  - Blind assessors: unclear. Not reported.  - Blind participants: Not possible. (Waitlist control)  - Blind therapists: Not possible.  - Intention-to-treat analysis: Yes. 22 patients in each group were initially enrolled, but two in each group were dropped-out and 20 patients in each group finished the study and analyzed. (Drop-out rate is low thus unlikely to change the robustness of study results.)  - Between group comparisons: Yes  - Funding: Not reported  - Setting: Community shelter for elderly, Seoul, South Korea. Subjects were recruited from Community shelter for elderly located in Seoul.  - Informed consents and ethics approval: Not reported |
| Participants | 44elderly insomnia patients enrolled, 40 patients finished the study.  Group 1: treatment group (males); 20 (7)  Group 2: control group (males); 20 (7)  Age: ranged from 65 to 74 (n=4), from 75-79 (n=8), and over 80 (n=8) (Group 1); ranged from 65-74 (n=6), from 75-79 (n=6) and over 80 (n=8) (Group 2)  Inclusion criteria: 1) Elderly patients aged over 65 with insomnia dwelling in Seoul 2) Ability to communicate with investigators 3) Understand the research purpose and agreed to participate 4) No sleep medication within one month.  Exclusion criteria: Not reported  Associated disease: Not reported  Duration of disorder: Not reported  Previous treatments: Not reported |
| Interventions | Group 1 (Treatment): Auricular acupressure treatment on the ear (Name of points: Calming down (鎭靜), Hundred spirit (百靈))  Group 2 (Control): No treatment. Waitlist.  Investigator pressed the ear studs to stimulate points.  Duration of treatment: Studs were replaced at a 3-day interval. Totally 5 sessions were provided. (Total treatment periods were 15 days)  Information on practitioners for acupuncture treatments: RN investigator trained for 4 weeks about auricular acupuncture by a traditional Korean medicine doctor. |
| Outcomes | Outcomes:  1. Oh’s sleeping score (ranged 15 to 60 scores; higher scores reflect better sleep).  2. Overall Self-satisfaction scale for sleep (ranged 0 to 10; higher scores reflect beneficial outcomes)  Table 3., Table 5.  Oh’s sleeping score: Treatment group: 22.05 ± 0.16 (baseline), 54.00 ± 0.24 (post-treatment); Control group: 23.35 ± 0.17 (baseline), 23.35 ± 0.0 (post-treatment)  Overall Self-satisfaction scale: Treatment group: 1.65 ± 0.49 (baseline), 8.45 ± 0.90 (post-treatment); Control group: 1.60 ± 0.50 (baseline), 1.53 ± 0.38 (post-treatment)  Complications: unclear (not reported for the occurrence of adverse event) |
| Notes | Language: Korean  Publication: Full paper  Dropouts: 2 in treatment and control group, respectively. No reasons described.  Duration of follow-up: 15 days (from baseline)  Additional information from authors: not sought  Conclusion: Auricular acupuncture was effective on insomnia in Korean elderly. |

**Assessment of Risk of Bias: Sok 2005 [11]**

| **Domain** | **Low risk of bias?**  Yes/No/Unclear* | | | **Description**  **(With Reasons of rating risk of bias)** |
| --- | --- | --- | --- | --- |
| **Was the allocation sequence adequately generated?** |  |  |  | Unclear. Not reported |
| **Was allocation adequately concealed?** |  |  |  | Unclear. Not reported |
| **Blinding: Participant** |  |  |  | Not possible (waitlist control) |
| **Attempt to confirm patient blinding for sham control** |  |  |  | Not applicable |
| **Blinding: Assessor** |  |  |  | Separate assessor was involved. However, whether this assessor was blinded remains unclear. |
| **Blinding: Other participants** |  |  |  | Not possible. |
| **Incomplete outcome report** |  |  |  | 22 patients in each group were initially enrolled, but two in each group were dropped-out and 20 patients in each group finished the study and analyzed. (Drop-out rate is low thus unlikely to change the robustness of study results.) |
| **Selective outcome reporting** |  |  |  | No study protocol was available. |
| **Other sources of bias** |  |  |  | Non-remarkable. |
| **Notes** |  |  |  |  |

5. Relevant review title: Acupuncture for tension-type headache

| Study information | Study ID: Kwak 2008 [12]  Title: Persisting effects of acupuncture method for chronic tension-type headache; a randomized controlled trial  Reference details: Journal of Korean Acupuncture and Moxibustion Society. 2008;25(2):165-177.  Correspondance: Hong Kwon-Eui.E-mail: [hkeacu@dju.ac.kr](mailto:hkeacu@dju.ac.kr); Lee Sang-bong. E-mail: sbneuro@medimail.co.kr |
| --- | --- |
| Methods | - Random allocation: Yes. Randomization table used.  - Concealed allocation: Yes. Telephone or e-mail notification of allocation results only to the acupuncture practitioner.  - Baseline comparability: Authors insisted that baseline characteristics were comparable in both groups, in terms of age, gender, onset time point of symptoms, duration of headache, frequency of headache per each week, intensity of headache and analgesic comsumption status.  - Blind assessors: Yes. Assessor did not know the group allocation result.  - Blind participants: Yes. (authors insisted that participants were well-blinded about the group allocation result and blinding credibility test confirmed successful blinding of participants)  - Blind therapists: Not possible. (However, TKM doctors who involved in the practice of study acupuncture in both groups were instructed to maintain minimal interaction with patients and not allowed to access the patient information except the allocation result).  - Dropouts/withdrawals: Unclear. Not reported whether dropouts/withdrawals were occurred. (analysis were based on 32 patients who completed the study)  - Between group comparisons: Yes  - Observation period: baseline period 3 days; treatment 4 weeks; post-treatment follow-up 12 weeks.  - Funding: Korea Institute of Oriental Medicine, 2007.  - Setting: Daejeon university hospital, Daejeon, South Korea. Subjects were recruited via hospital and university webpage postings, local community newspapers and poster.  - Informed consents and ethics approval: Both obtained. |
| Participants | The number of initially enrolled patients was not reported. 32 patients finished the study.  Condition: tension-type headache (HIS)  Demographics: mean age 45 and 48 years in treatment and control group, respectively.  Time since onset of headaches: onset age of headache was 30 and 36 years in treatment and control group, respectively. |
| Interventions | Acupuncture points: semi-fixed regimen. Selection of points was individually chosen according to symptoms.  Information on acupuncturists: not reported  De-qi achieved?: not reported  Number of treatment sessions: 8  Frequency of treatment sessions: 2/week  Control interventions: penetrating sham needle treatment on non-acupuncture points. Totally 6 points used. |
| Outcomes | Methods for outcome measures: diary and questionnaires  Outcomes: pain intensity (Visual analogue scale), Headache Disability intensity, Six-point likert scale, Pain pressure thresholds (by algometry), safety of acupuncture. Complications: No adverse events occurred in both groups. |
| Notes | Language: Korean  Publication: Full paper  Dropouts: Unclear.  Duration of follow-up: 16 weeks (from baseline)  Additional information from authors: not sought  Conclusion: Acupuncture treatment has persisting effects that improve the symptoms and decrease the temporal muscle tenderness in patient with chronic tension-type headache. |

**Assessment of Risk of Bias: Kwak 2008 [12]**

| **Domain** | **Low risk of bias?**  Yes/No/Unclear* | | | **Description**  **(With Reasons of rating risk of bias)** |
| --- | --- | --- | --- | --- |
| **Was the allocation sequence adequately generated?** |  |  |  | Yes. Randomization table used. |
| **Was allocation adequately concealed?** |  |  |  | Yes. Telephone or e-mail notification of allocation results only to the acupuncture practitioner. |
| **Blinding: Participant** |  |  |  | Yes. (authors insisted that participants were well-blinded about the group allocation result and blinding credibility test confirmed successful blinding of participants) |
| **Attempt to confirm patient blinding for sham control** |  |  |  | Yes |
| **Blinding: Assessor** |  |  |  | Yes. Assessor did not know the group allocation result. |
| **Blinding: Other participants** |  |  |  | Not possible. |
| **Incomplete outcome report** |  |  |  | Dropouts/withdrawals: Unclear. Not reported whether dropouts/withdrawals were occurred. (analysis were based on 32 patients who completed the study) |
| **Selective outcome reporting** |  |  |  | Some of outcomes (Headache disability index and six-point likert scale) were not reported as scores, but only statistical significance with p-values were reported. |
| **Other sources of bias** |  |  |  | Non-remarkable. |
| **Notes** |  |  |  |  |

6. Relevant review title: Acupuncture for neck disorders

| Study information | Study ID: Kwak 2012 [13]  Title: Acupuncture for whiplash injury – a feasibility pilot study-  Reference details: Master dissertation. Kyung Hee University 2011. (Acupuncture for whiplash-associated disorder: a randomized, waiting-list controlled, pilot trial. Eur J Integr Med 2012;4(2):e151-e158.)  Correspondance: Do-Young Choi. E-mail: choi4532@unitel.co.kr |
| --- | --- |
| Methods | - Random allocation: Yes. A computer-generated randomization table used.  - Concealed allocation: Unclear. Authors insisted that group allocation was concealed by using sealed envelopes. However, whether those envelops were opaque or not remains unclear.  - Intention-to-treat: Yes.  - Baseline comparability: Baseline characteristics were comparable in both groups, in terms of age, gender, body mass index, duration to onset and baseline outcome scores.  - Blind assessors: Unclear. Not reported.  - Blind participants: Not possible (waitlist control)  - Blind therapists: Not possible  - Dropouts/withdrawals: 3 patients dropped-out. Reasons include minimal treatment effects or mild adverse events (n=2) and oversea-travel during the trial (n=1)  - Power analysis: not conducted because this study was a pilot trial.  - Funding: University of Tsukuba, Japan.  - Setting: Kyung Hee Oriental Medical Center, Seoul, South Korea.  - Informed consents and ethics approval: Both obtained. |
| Participants | - chronic whiplash-associated disorder with cervical symptoms continuing for 3 months or more  - Recruitment: Subjects were recruited through advertisements in local newspapers and on the homepage of the hospital from December 8, 2009 to October 14, 2010. |
| Interventions | Index treatment:  Group 1:  Acupuncture points: A range of acupuncture points to treat symptoms were discussed by the Korean and Japanese acupuncture experts with the clinical experience and the literature review. 10 local points and other distal points were recommended in the consensus. Used points at least once included SI15, SI14, BL10, BL12, BL13, BL14, GB20, GB21 and TE15 as local points and SI2, SI3, SI5, SI7, LI11, BL60, BL62, BL66, GB40, GB41 and TE5 as distal points.  Depth of needling: 1-2 cm perpendicularly.  Manipulation methods: manual rotation to elicit De-qi sensation.  Needle retention time: 15 min  Information on acupuncturists: a licensed oriental medicine doctor with at least 3 years of clinical experience after a 6-year course in oriental medicine education.  Comparison treatment 1: No treatment (waitlist control)  Co-intervention: Usual care including physiotherapy, exercise and rest. Both groups were instructed to stop to taking analgesics and anti-inflammatory agents for pain relief during the trial period.  Number of treatment sessions: 6  Frequency of treatment sessions: 3/week  Duration of treatments: 2 weeks  Duration of follow-up: 2 weeks (outcomes were measured after the end of final treatment). |
| Outcomes | Pain intensity: (VAS 10 point scale: 0 to no pain and 10 to extreme pain)  Baseline mean: acupuncture 4.59, waitlist 4.88  End of study mean: acupuncture 2.74, waitlist 4.47  Absolute benefit: acupuncture 1.84, waitlist 0.41  Range of motion: (Cervical flexion, extension, left and right lateral bending, let and lateral rotation)  Quality of life and function: (SF-36 scores)  Psychological change: (Self-Rating Depression scale, Cornell Medical Intex)  Reported results: Patients with whiplash-associated disorder received six sessions of acupuncture therapy during 2 weeks, and this treatment resulted in a significant decrease in pain intensity compared to the control group.  Complications: No adverse events occurred in both groups. Mild, transient adverse events were occurred in three patients (bruising in two, fatigue in one).  Cost of care: not reported |
| Notes | Language: Korean (Master dissertation), English (the article published in journal)  Publication: Full paper  Additional information from authors: not sought  Conclusion: Patients with whiplash-associated disorder received six sessions of acupuncture therapy during 2 weeks, and this treatment resulted in a significant decrease in pain intensity compared to the control group. |

**Assessment of Risk of Bias: Kwak 2011[13]**

| **Domain** | **Low risk of bias?**  Yes/No/Unclear* | | | **Description**  **(With Reasons of rating risk of bias)** |
| --- | --- | --- | --- | --- |
| **Was the allocation sequence adequately generated?** |  |  |  | Yes. A computer-generated randomization table used. |
| **Was allocation adequately concealed?** |  |  |  | Unclear. Authors insisted that group allocation was concealed by using sealed envelopes. However, whether those envelops were opaque or not remains unclear. |
| **Blinding: Participant** |  |  |  | Not possible. (waitlist control) |
| **Attempt to confirm patient blinding for sham control** |  |  |  | Not applicable |
| **Blinding: Assessor** |  |  |  | Unclear. Not reported. |
| **Blinding: Other participants** |  |  |  | Not possible. |
| **Incomplete outcome report** |  |  |  | ITT analyzed |
| **Selective outcome reporting** |  |  |  | All measured outcomes were well-reported. Protocol is available (NCT01395511) |
| **Other sources of bias** |  |  |  | Non-remarkable. |
| **Notes** |  |  |  |  |

7. Relevant review title: Stimulation of the wrist acupuncture point P6 for preventing postoperative nausea and vomiting

| Study information | Study ID: Kim 2004 [14]  Title: Transcutaneous electrical stimulation of the P6 acupoint reduces postoperative nausea after minor breast surgery.  Reference details: Korean J Anesthesiol 2004;47(6):834-839.  Correspondance: Soon Im Kim. E-mail: soonim@hosp.sch.ac.kr |
| --- | --- |
| Methods | - Random allocation: Unclear. No randomization methods reported.  - Concealed allocation: Unclear.  - Intention-to-treat: Yes.  - Baseline comparability: Baseline characteristics were comparable in both groups, in terms of age, duration of anesthesia, previous surgery history, previous PONV history.  - Blind assessors: Yes. A research investigator who did not know the allocation results interviewed the patients both at baseline and post-operative evaluation.  - Blind participants: Yes. Placebo controlled trial.  - Blind therapists: Unclear. Whether research investigators who provided the real and placebo relief-bands were aware of the group allocation results were not reported.  - Dropouts/withdrawals: None.  - Funding: Not reported.  - Setting: Recovery room for surgery and inpatient ward, Soonchunhyang University hospital, Seoul, Korea.  - Informed consents and ethics approval: Not reported. |
| Participants | - 66 women classified as American Society of Anesthesiology Classification I or II class, undergoing minor breast surgery under general anesthesia using sevoflurane.  - Exclusion criteria:1) patients with respiratory, circulatory or brain diseases 2) liver and kidney abnormalities 3) nausea or vomiting before 24-hour from the due time of surgery or received antiemetics 4) pregnant women 5) excessively obese patients |
| Interventions | Electrical acupressure band (ReliefBand) placed on PC6 point 10 minutes before the surgery and left on for 24 hour. Bi- or Unilateral placement of band was not reported.  Electrical stimulation: 35 mA  Reference group: Mock electrical acupressure band which were unpowered. |
| Outcomes | Incidence of nausea and vomiting (6 and 24 hour post-surgery).  Antiemetic requirements (6 and 24 hour post-surgery)  Type of antiemetics: intravenous Ondansetron 4 mg as needed by the patient.  Complications: Not reported about the occurrence of adverse events. |
| Notes | Language: Korean  Publication: Full paper  Additional information from authors: not sought  Conclusion: Transcutaneous electrical stimulation of the P6 acupoint significantly reduced the incidence and severity of nausea, but not the incidence and seerity of vomiting, for female patients undergoing minor breast surgery during the first 24 hour after surgery. |

**Assessment of Risk of Bias: Kim 2004 [14]**

| **Domain** | **Low risk of bias?**  Yes/No/Unclear* | | | **Description**  **(With Reasons of rating risk of bias)** |
| --- | --- | --- | --- | --- |
| **Was the allocation sequence adequately generated?** |  |  |  | Unclear. No randomization methods reported. |
| **Was allocation adequately concealed?** |  |  |  | Unclear. Not reported. |
| **Blinding: Participant** |  |  |  | Yes. Placebo controlled trial. |
| **Attempt to confirm patient blinding for sham control** |  |  |  | Unclear. Not reported. |
| **Blinding: Assessor** |  |  |  | Yes. A research investigator who did not know the allocation results interviewed the patients both at baseline and post-operative evaluation. |
| **Blinding: healcare providers** |  |  |  | Unclear. Whether research investigators who provided the real and placebo relief-bands were aware of the group allocation results were not reported. |
| **Incomplete outcome report** |  |  |  | ITT analyzed |
| **Selective outcome reporting** |  |  |  | All measured outcomes were well-reported. |
| **Other sources of bias** |  |  |  | Non-remarkable. |
| **Notes** |  |  |  |  |

| Study information | Study ID: Kim 2001 [15]  Title: Transcutaneous electrical acupoint stimulation of prevention of postoperative nausea and vomiting in female patients receiving iv-PCA.  Reference details: Intraveous Anesthesia 2001;5(4):210-215. (journal names translated)  Correspondance: Soon Im Kim. E-mail: soonim@hosp.sch.ac.kr |
| --- | --- |
| Methods | - Random allocation: Unclear. No randomization methods reported.  - Concealed allocation: Unclear.  - Intention-to-treat: Yes.  - Baseline comparability: Baseline characteristics were comparable in both groups, in terms of age, body weight, duration of anesthesia and previous PONV history.  - Blind assessors: Yes. A research investigator who did not know the allocation results interviewed the patients both at baseline and post-operative evaluation.  - Blind participants: Yes. Placebo controlled trial.  - Blind therapists: Unclear. Whether research investigators who provided the real and placebo relief-bands were aware of the group allocation results were not reported.  - Dropouts/withdrawals: None.  - Funding: Not reported.  - Setting: Recovery room for surgery and inpatient ward, Soonchunhyang University hospital, Seoul, Korea.  - Informed consents and ethics approval: Not reported. |
| Participants | - 60 women classified as American Society of Anesthesiology Classification I or II class, undergoing major orthopedic surgery under general anesthesia using an isoflurane or enflurane as well as being aware of how to use the intravenous patient-controlled analgesia .  - Exclusion criteria:1) patients with respiratory or circulatory diseases 2) liver and kidney abnormalities 3) nausea or vomiting before 24-hour from the due time of surgery or received antiemetics 4) pregnant women 5) excessively obese patients 6) history of drug addiction |
| Interventions | Electrical acupressure band (ReliefBand) placed on PC6 point 10 or 20 minutes before the surgery and left on for 24 hour. Bi- or Unilateral placement of band was not reported.  Electrical stimulation: simulation parameters not reported  Reference group: Mock electrical acupressure band which were unpowered. |
| Outcomes | Incidence of nausea and vomiting (6 and 24 hour post-surgery).  Antiemetic requirements (6 and 24 hour post-surgery)  Type of antiemetics: intravenous Ondansetron 4 mg as needed by the patient.  Complications: Not reported about the occurrence of adverse events. |
| Notes | Language: Korean  Publication: Full paper  Additional information from authors: not sought  Conclusion: Transcutaneous electrical stimulation of the P6 acupoint does not reduce the incidence and severity of PONV in female patients receiving the fentanyl iv-PCA during the first 24 hour after surgery. |

**Assessment of Risk of Bias: Kim 2001 [15]**

| **Domain** | **Low risk of bias?**  Yes/No/Unclear* | | | **Description**  **(With Reasons of rating risk of bias)** |
| --- | --- | --- | --- | --- |
| **Was the allocation sequence adequately generated?** |  |  |  | Unclear. No randomization methods reported. |
| **Was allocation adequately concealed?** |  |  |  | Unclear. Not reported. |
| **Blinding: Participant** |  |  |  | Yes. Placebo controlled trial. |
| **Attempt to confirm patient blinding for sham control** |  |  |  | Unclear. Not reported. |
| **Blinding: Assessor** |  |  |  | Yes. A research investigator who did not know the allocation results interviewed the patients both at baseline and post-operative evaluation. |
| **Blinding: healcare providers** |  |  |  | Unclear. Whether research investigators who provided the real and placebo relief-bands were aware of the group allocation results were not reported. |
| **Incomplete outcome report** |  |  |  | ITT analyzed |
| **Selective outcome reporting** |  |  |  | All measured outcomes were well-reported. |
| **Other sources of bias** |  |  |  | Non-remarkable. |
| **Notes** |  |  |  |  |

8. Relevant review title: Acupuncture for cancer pain in adults

| Study information | Study ID: Yoo 2008 [16]  Title: The effect of sweet bee venom pharmacopuncture (SBVP) on cancer-related pain: a randomized controlled trial and double blinded – pilot study.  Reference details: Journal of Pharmacopuncture 2008;11(1):21-29.  Correspondance: Hwa-Seung Yoo. E-mail: altyhs@dju.kr |
| --- | --- |
| Methods | - RCT, 2-arm  - Random allocation: Yes. A randomization table used.  - Concealed allocation: Unclear.  - Intention-to-treat: No. only patients who finished the trial were analyzed.  - Baseline comparability: unclear. (unable to calculate statistical comparability due to small sample size)  - Blind assessors: Unclear. The assessor was separated from the acupuncture practitioner. However, whether the assessor was aware of the group allocation results was not reported.  - Blind participants: Yes. Authors insisted that participants were successfully blinded to their allocated treatments.  - Blind therapists: Yes. Acupuncture practitioners received the injection needle by an research associate. Injections were identical in terms of appearance. Acupuncture practitioner was blinded to the group allocation results. (authors insisted).  - Dropouts/withdrawals: Worsened pain (n=1; treatment group), No effects (n=1; control group)  - Funding: Korean Pharmacopuncture Institute, 2007.  - Setting: East-West Cancer Center of Dunsan Oriental Hospital, Daejeon University, Daejeon, South Korea.  - Informed consents and ethics approval: Informed consents about the sweet bee venom acupuncture treatment (unclear about the clinical trial), Ethics approval unclear (not reported) |
| Participants | - 11 participants with cancer pain ranomised to 2 groups: Group 1 (treatment group using the Sweet bee venom acupuncture), Group 2 (control group using normal saline injection on acupuncture points) |
| Interventions | Group 1: Sweet bee venom acupuncture on CV12. 1 cc injection. Bee venom diluted into 0.1mg/ml.  Group 2: normal saline injected on CV 12. 1cc injection.  Duration, session and frequency of treatments: once daily for 5 consecutive days.  A disposable injector (1cc, 28 gauge; Samwoo, Inc, Korea) was used. |
| Outcomes | - Average pain measured at each immediate post-treatment  - Pain in last 24 hours measured  Measured at: each pre- and post-intervention.  Complications: Not reported about the occurrence of adverse events. |
| Notes | Language: Korean  Publication: Full paper  Additional information from authors: not sought  Conclusion: SBVP shows potential as an effective treatment for immediate relief of cancer-related pain, although further study will be needed on the large scale. |

**Assessment of Risk of Bias: Yoo 2008 [16]**

| **Domain** | **Low risk of bias?**  Yes/No/Unclear* | | | **Description**  **(With Reasons of rating risk of bias)** |
| --- | --- | --- | --- | --- |
| **Was the allocation sequence adequately generated?** |  |  |  | Yes. A randomization table used. |
| **Was allocation adequately concealed?** |  |  |  | Unclear. Not reported. |
| **Blinding: Participant** |  |  |  | Yes. Authors insisted that participants were successfully blinded to their allocated treatments. |
| **Attempt to confirm patient blinding for sham control** |  |  |  | Unclear. Not reported. |
| **Blinding: Assessor** |  |  |  | Yes. A research investigator who did not know the allocation results interviewed the patients both at baseline and post-operative evaluation. |
| **Blinding: healcare providers** |  |  |  | Yes. Acupuncture practitioners received the injection needle by an research associate. Injections were identical in terms of appearance. Acupuncture practitioner was blinded to the group allocation results. (authors insisted). |
| **Incomplete outcome report** |  |  |  | Patients who finished the study were analyzed. Reasons of attrition may affect the robustness of study result (worsened symptoms, no treatment effects). |
| **Selective outcome reporting** |  |  |  | Outcomes were poorly defined. Table 3 (pain intensity in Group 1) and 4 (pain intensity in Group 2) is duplicated. High risk of bias is suspected. |
| **Other sources of bias** |  |  |  | Poor reporting of intervention and methodology. Too small sample size. |
| **Notes** |  |  |  |  |

**References of 16 included Korean studies**

1. Kwon Y-D, Lee S-G, Lee C-W, Jung S-K, Kim D-E, et al. (2007) The short-term efficacy of acupuncture for chronic low back pain: randomized sham controlled trial. Journal of Oriental Rehabilitation Medicine 17: 123-132.

2. Lee J-B, Im J-G, Lee H-G, Kim J-U, Yook T-H, et al. (2011) The comparison of effectiveness between acupuncture and its cotreatment with Wan-Gwa acupuncture on the treatment of low back pain. Journal of Korean Acupuncture and Moxibustion society 28: 43-47.

3. Kim S-J, Lee H, Jung H-S, Kim E-S, Woo J-H, et al. (2010) A clinical study on effect of electro-acupuncture treatment for lumbago patients caused by traffic accident. Journal of Korean Acupuncture & Moxibustion Society 27: 117-123.

4. Lee Y-K, Park S-Y, Jeon H-J, Kim S-H, Kim J-H, et al. (2009) The comparative study on effect of body acupuncture and 8 constitutional acupuncture in acute stage lumbago - for patients checked up the Pancreotonia -. Journal of Korean Acupuncture and Moxibustion Society 26: 181-188.

5. Min K-O, Kim S-H, Park S-J (2002) The effects of the needle electrode electrical simulation in the auricular therapy on the smokers and non-smokers with low back pain. Journal of Korean Physical Therapist [translated] 9: 45-52.

6. Nam D-W, Lim S, Kim J-I, Kim K-S, Lee D-I, et al. (2007) Clinical observation of acupuncture and nerve block treatment for adhesive capsulitis patients. Journal of Korean Acupuncture and Moxibustion society 24: 159-171.

7. Nam D-W, Choi Y-S, Kim H-B, Kim J-I, Lim S, et al. (2007) Randomized controlled trial of East-West collaborate medical treatment on female chronic shoulder pain patients. Journal of Korean Acupuncture and Moxibustion Society 24: 113-122.

8. Lee H, Hong K-E, Kim Y-I, Yim Y-K, Ahn T-W, et al. (2006) A clinical trial of acupuncture treatment for frozen shoulder. Journal of Korean Acupuncture and Moxibustion Society 23: 165-177.

9. Yoon H-M, Kim C-H, Park J-H, Han M-S, Lee I-S, et al. (2008) Effect of acupuncture treatment on the primary dysmenorrhea (a study of single blind, sham acupuncture, randomized, controlled clinical trial). Journal of Korean Acupuncture and Moxibustion Society 25: 139-156.

10. Jung I-S, Kim JS, Yoo H-S (2010) Effect of auricular acupress therapy on insomnia of cancer patients: randomized, single blinded, placebo controlled trial. Journal of Pharmacopuncture 13: 93-100.

11. Sok SH, Kim KB (2005) Effects of auricular acupuncture on insomnia in Korean elderly. J Korean Acad Nurs 35: 1014-1024.

12. Kwak B-M, Kim M-JK, Y-M, Lee J-M, Park Y-C, Jo J-H, et al. (2008) Persisting effects of acupuncture method for chronic tension-type headache; a randomized controlled trial. Journal of Korean Acupuncture and Moxibustion Society 25: 165-177.

13. Kwak HY Acupuncture for whiplash injury - a feasibility pilot study - Master Dissertation. Kyung Hee University 2011.

Kwak H-Y, Kim J-I, Park, J-M, Lee S-H, Yoo H-S, et al. (2012) Acupuncture for whiplash-associated disorder: a randomized, waiting-list controlled, pilot trial. Eur J Integr Med 4:e151-e158.

14. Kim SI, Yoo IS, Park HN, Ok SY, Kim SC (2004) Transcutaneous electrical stimulation of the P6 acupoint reduces postoperative nausea after minor breast surgery. Korean J Anesthesiol 47: 834-839.

15. Kim SI, Kim SC, Choi JK, Jin HC (2001) Transcutaneous electrical acupoint stimulation of prevention of postoperative nausea and vomiting in female patients receiving iv-PCA. Intraveous Anesthesia [Translated] 5: 210-215.

16. Yoo H-S, Kim J-S (2008) The effect of sweet bee venom pharmacopuncture (SBVP) on cancer-related pain: a randomized controlled trial and double blinded – pilot study. . Journal of Pharmacopuncture 11: 21-29.
