# Supplementary material for: Impact of Including Korean Randomized Controlled Trials in Cochrane Reviews of Acupuncture
Source: PLoS One. 2012 Oct 11;7(10):e47619. doi: 10.1371/journal.pone.0047619 (PMC3469498; doi:10.1371/journal.pone.0047619)
Supplement: Table S2 — Characteristics of 99 excluded Korean studies. (DOC) [file pone.0047619.s002.doc]

Table S2. Characteristics of 99 excluded Korean studies

| Conditions | Reference number | First Author | Reason for exclusion |
| --- | --- | --- | --- |
| Low back pain | [1] | Moon 2001 | Back pain with or without referral to proximal extremity |
|  | [2] | Lee 2007 | LBP with sciatica |
|  | [3] | Chang 2002 | Lumbar disc herniation |
|  | [4] | Kim 2008 | Bee Venom acupuncture used |
|  | [5] | Lee 2007a | Bee Venom acupuncture used |
|  | [6] | Lee 2007b | Lumbar disc herniation |
|  | [7] | Ko 2005 | Studied on healthy individuals |
|  | [8] | Yu 2006 | Lumbar disc herniation |
|  | [9] | Chae 2001 | Lumbar disc herniation |
|  | [10] | Chung 2003 | Lumbar disc herniation |
|  | [11] | Ahn 2002 | more than 60% (12/19) had specific pathological entities (disc herniation, degenerative intervertebral disc, spondylolisthesis) |
|  | [12] | Ryu 2001 | majority of patients (24/25) had specific pathological entities (disc herniation, spinal stenosis, compression fracture) |
|  | [13] | Kim 2005 | Bee Venom acupuncture used |
|  | [14] | Yoon 2010 | Lumbar disc herniation |
|  | [15] | Lee 2006 | Sciatica |
|  | [16] | Lee 1996 | Conditions combined (LBP and neck) |
| Shoulder pain | [17] | Lee 2007 | MPS shoulder pain |
|  | [18] | Nam 2006a | duplicated publication |
|  | [19] | Kim 2007 | duplicated publication |
|  | [20] | Nam 2006b | duplicated publication |
| Insomnia | [21] | Sok 2000 | not a RCT |
| Prevention of PONV | [22] | Kim 1996 | not PC6 stimulation |
|  | [23] | Lee 2007 | control: no waiting (not eligible for PONV review) |
| Tension-type headache | [24] | Hong 2007 | Study period less than 8 weeks (2-week therapy + 1-week follow-up) |
| Neck disorders | [25] | Lee 2003 | Not a traditional needle acupuncture (acupoint injection as a primary intervention) |
|  | [26] | Joung 2010 | Not a neck pain (headache due to whiplash injury) |
|  | [27] | Song 2007 | Not a traditional needle acupuncture (acupoint injection as a primary intervention) |
|  | [28] | Seol 2005 | Not a traditional needle acupuncture (acupoint injection as a primary intervention) |
|  | [29] | Cho 2006 | for healthy individuals |
|  | [30] | Park 2011 | Comparing two acupuncture techniques |
|  | [31] | Lee 2008 | Comparing two acupuncture techniques |
|  | [32] | Kim 2005 | Not a traditional needle acupuncture (acupoint injection as a primary intervention) |
|  | [33] | Kang 2002 | Not a traditional needle acupuncture (acupoint injection as a primary intervention) |
|  | [34] | Kim 2002 | Not a traditional needle acupuncture (acupoint injection as a primary intervention) |
| Primary dysmenorrhea | [35] | Lee 2003 | already included in Cochrane review |
|  | [36] | Cho 2007 | Acupuncture is not a intervention of primary interest. Traditional herbal medicine was compared with traditional herbal medicine plus acupuncture. |
|  | [37] | Han 2009 | Duplicated publication. Unpublished manuscript for Master degree |
|  | [38] | Kim 2008 | The trial injected placenta extraction into the acupuncture point, this intervention did not meet the inclusion criteria |
|  | [39] | Lee 2007 | No information about duration of dysmenorrhea was available. |
|  | [40] | Yuk 2005 | Information whether true randomization or not reported. No information about duration of dysmenorrhea was available. |
| Peripheral OA | [41] | Lee 2005 | Not a traditional needle acupuncture (acupressure with capsicum plaster) |
|  | [42] | Na 2007 | Not a traditional needle acupuncture (acupoint injection) |
|  | [43] | Min 2009 | Study observation periods less than 6 weeks |
|  | [44] | Ryu 2004 | No description on randomization (random sampling, but no information regarding allocation methods) |
|  | [45] | Kim 2006 | comparing different acupuncture treatment programs (individualized versus standardized) |
|  | [46] | Lee 2003 | Not a traditional needle acupuncture (acupoint injection) |
|  | [47] | Seo 2008 | Not true randomization (allocated by visiting date) |
|  | [48] | Park 2006 | Comparing different acupuncture treatment programs: acupoint injection versus needle acupuncture |
|  | [49] | Kim 2009 | Not a traditional needle acupuncture (acupoint injection) |
|  | [50] | Kim 2008 | Not a traditional needle acupuncture (acupoint injection) |
|  | [51] | Park 2008 | comparing different acupuncture treatment programs (local points versus distal points) |
|  | [52] | Byun 2007 | comparing different acupuncture treatment programs (individualized versus standardized) |
|  | [53] | Min 2008 | Comparing different acupuncture treatment programs: warm needling with moxa versus needle acupuncture |
|  | [54] | Chae 2004 | comparing different acupuncture treatment programs (constitutional acupuncture versus body acupuncture) |
|  | [55] | Yang 2008 | Comparing different acupuncture treatment programs: acupoint injection versus warm needling |
|  | [56] | An 2006 | Comparing different acupuncture treatment programs: acupoint injection versus needle acupuncture |
| Lateral elbow pain | [57] | An 2004 | Randomized controlled trial of 2 different methods of acupuncture. Not included in the review as not comparing acupuncture to placebo or another intervention. |
| Smoking Cessation | [58] | Chae 2009 | Condition is not a smoking withdrawal syndrome |
| Bell’s palsy | [59] | Kim 2004 | No description on randomization |
|  | [60] | Kim 2001 | No description on randomization (random sampling, but no information regarding allocation methods) |
|  | [61] | Kim 2010 | comparing different acupuncture treatment programs |
|  | [62] | Lee 2004 | No description on randomization (random sampling, but no information regarding allocation methods) |
|  | [63] | Park 2010 | comparing different acupuncture treatment programs |
|  | [64] | Choi 2010 | comparing different acupuncture treatment programs |
|  | [65] | Shin 2009 | Acupuncture was applied in both groups (routine ward care in TKM hospital plus acupoint injection versus routine care alone) |
|  | [66] | An 2005 | comparing different acupuncture treatment programs (electroacupuncture vs manual acupuncture) |
|  | [67] | Chun 2009 | No true randomization (allocated by admission date) |
|  | [68] | Choi 2009a | comparing different acupuncture treatment programs; acupuncture was applied in each group |
|  | [69] | Choi 2009b | Acupuncture was applied in both groups (routine ward care in TKM hospital plus acupoint injection versus routine care alone) |
|  | [70] | Hwang 2006 | Acupuncture was applied in both groups (acupuncture plus energy therapy versus acupuncture alone) |
| Stroke rehabilitation | [71] | Lee 2007 | The duration of stroke since onset was not stated clearly in the trial; it was not possible to include data from this trial in the analysis |
|  | [72] | Lee 2005 | The trial aimed to assess effects of two methods of acupuncture on subacute or chronic stroke. Acupoints were different between the two groups. |
|  | [73] | Cho 2006 | The trial included patients with acute stroke (less than one month since onset).  Confounded (general acupuncture intervention were adopted for routine rehabilitation care). |
|  | [74] | Cho 2005 | The trial aimed to assess effects of two methods of pharmacopuncture. Acupoints were the same between the two groups.  Confounded (general acupuncture intervention were adopted for routine rehabilitation care). |
|  | [75] | Kim 2005 | Confounded (the trial aimed to assess the effects of the combination of acupuncture on specific interosseous area of hand, classic acupuncture, electroacupuncture and classic acupuncture, electroacupuncture). |
|  | [76] | Lee 2006 | Confounded (general acupuncture intervention were adopted for routine rehabilitation care). |
|  | [77] | Seo 1998 | The duration of stroke since onset was not stated in the trial; it was not possible to include data from this trial in the analysis |
|  | [78] | Eom 2009 | The duration of stroke since onset was not stated in the trial; it was not possible to include data from this trial in the analysis  The trial aimed to assess effects of two methods of acupuncture on subacute or chronic stroke. Acupoints were the same between the two groups. |
|  | [79] | Ko 2007 | The duration of stroke since onset was not stated clearly in the trial; it was not possible to include data from this trial in the analysis |
|  | [80] | Choi 2003 | The duration of stroke since onset was not stated clearly in the trial; it was not possible to include data from this trial in the analysis |
|  | [81] | Lee 1997 | The trial included patients with acute stroke (less than one month since onset).  The trial aimed to assess effects of two methods of acupuncture on subacute or chronic stroke. Acupoints were different between the two groups. |
|  | [82] | Cho 2002 | The trial included patients with acute stroke (less than one month since onset).  The trial aimed to assess effects of two methods of acupuncture on subacute or chronic stroke. Acupoints were different between the two groups. |
|  | [83] | Ryu 2006 | The trial aimed to assess effects of two methods of acupuncture (compare high frequency electroacupuncture and low frequency electroacupuncture). Confounded (general acupuncture intervention were adopted for routine rehabilitation care). |
|  | [84] | Bae 2004 | The trial included patients with acute stroke (less than one month since onset).  Confounded (general acupuncture intervention were adopted for routine rehabilitation care). |
|  | [85] | Sim 2003 | The trial aimed to assess effects of two methods of acupuncture (compare the effect between Yin and Yang meridians electroacupuncture). The trial included patients with acute stroke (less than one month since onset). |
|  | [86] | Kim 2008 | The duration of stroke since onset was not stated clearly in the trial; it was not possible to include data from this trial in the analysis Confounded (general acupuncture intervention were adopted for routine rehabilitation care). |
|  | [87] | Noh 2010 | Confounded (general acupuncture intervention were adopted for routine rehabilitation care). |
|  | [88] | Ryu 2001 | duplicated publication |
|  | [89] | Noh 2009 | Confounded (general acupuncture intervention were adopted for routine rehabilitation care). |
|  | [90] | Gwak 2009 | The duration of stroke since onset was not stated clearly in the trial; it was not possible to include data from this trial in the analysis. The trial primarily aimed to assess the effects of acupuncture on the pain control in patients with CPSP after stroke. |
|  | [91] | Kim 2000 | The trial included patients with acute stroke (less than one month since onset).  Confounded (the trial aimed to assess effects of electro-acupuncture compared with other active controls (FES, NMES)). |
|  | [92] | Lee 2004 | The duration of stroke since onset was not stated clearly in the trial; it was not possible to include data from this trial in the analysis No outcome measures of interest were available in the trial, which aimed to assess the effects of acupuncture for insomnia after stroke. Confounded (general acupuncture intervention were adopted for routine rehabilitation care). |
|  | [93] | Cho 2003 | The trial aimed to assess the effects of combination of body and scalp acupuncture compared with body acupuncture only.  The trial included patients with acute stroke (less than one month since onset). |
| Acute Stroke | [94] | Seo 2001a | The trial aimed to assess effects of two kinds of acupuncture on acute stroke (compare contralateral acupuncture with ipsilateral acupuncture). |
|  | [95] | Seo 2001b | The trial aimed to assess effects of two kinds of acupuncture on acute stroke (compare contralateral acupuncture with ipsilateral acupuncture). |
|  | [96] | Kim 2007 | The trial aimed to assess effects of two kinds of acupuncture on acute stroke (compare high frequency electroacupuncture and low frequency electroacupuncture). Confounded (general acupuncture intervention were adopted for routine rehabilitation care). |
|  | [97] | Ryu 2002 | Confounded (general acupuncture intervention were adopted for routine rehabilitation care). |
| Dysphagia in acute stroke | [98] | Yang 2008 | Different methods of electro-stimulation on acupoints were compared without a control group of placebo, sham or no intervention  The trial included patients with subacute or chronic stroke (more than one month since onset). |
| CINV | [99] | Seong 2010 | Not explicitly stated randomization (non-equivalent control group pre-post test design) |

LBP: low back pain; MPS: myofascial pain syndrome; RCT: randomized controlled trial; PONV: postoperative nausea and vomiting; OA: osteoarthritis; TKM: traditional Korean medicine; CPSP: central poststroke pain; FES: functional electrical stimulation; NMES: neuromuscular electrical stimulation; CINV; chemotherapy induced nausea and vomiting

**References of excluded Korean studies**

1. Moon S-E (2001) A New Approach to the Whole Body Intervention Program (General Coordinative Manipulation Program) of Nonspecific Back Disorder. J Kor Phys Ther Sci 15: 112-128.

2. Lee J-W, Kim D-I (2007) A Randomized Controlled Trial to Verify Effects of Kuesu Point on Low Back Pain and Accompanied Sciatica. Kor J Oriental Med 28: 86-94.

3. Chang B-S, Jin K-S, Kim J-W, Yang M-B, Kim I-D, et al. (2002) Clinical Study On The Remedial Effect of Oriental Medicine Used EAV(Meridian). J Kor Acup Mox Soc 19: 80-96.

4. Kim J-H, Jang S-H, Yoon H-M, Jang K-J, Ahn C-B, et al. (2008) The comparison of Effectiveness between Bee Venom and Sweet Bee Venom Therapy on Chronic Lower Back Pain. J Pharmacopuncture 11: 15-24.

5. Lee T-H, Hwang H-S, Chang S-Y, Cha J-H, Jung K-H, et al. (2007) The Comparison of Effectiveness between Bee Venom and Sweet Bee Venom Therapy on Low back pain with Radiating pain. J Pharmacopuncture 10: 85-89.

6. Lee S-H, Kang M-W, Lee H, Lee S-Y (2007) Effectiveness of Bee-venom Acpuncture and Ouhyul Herbal Acpuncture in Herniation of Nucleus Pulposus-comparison with Acpuncture Therapy Only. J Kor Acup Mox Soc 24: 197-205.

7. Ko S-K, Song H-G, Kim E-M, Park Y-S, Song M-S, et al. (2005) The Effect of Acupuncture Treatment on the Lumbar Extensor Muscle Flexibility. J Kor Acup Mox Soc 22: 79-90.

8. Yu S-M, Lee J-Y, Kwon K-R, Lee H-S (2006) Comparative Study of Acupuncture, Bee Venom Acupuncture, and Bee Venom Pharmacopuncture on the Treatment of Herniation of Nucleus Pulpous. J Kor Acup Mox Soc 23: 39-54.

9. Chae S-J, Kim N-O, Park Y-C, Son S-S (2001) Comparison of the Improvement of Subjective Symptoms between Body Acupuncture Group & 8 Constitution Acupuncture Group. J Kor Acup Mox Soc 18: 48-55.

10. Chung W-S, Lee J-S, Chung S-H, Kim S-S (2003) The Effect of Bee Venom Acupuncture on Patient with Herniation of Nucleus Pulposus of Lumbar Spine. J Oriental Rehab Med 2003: 2.

11. Ahn J-L, Lee I-S, Cha S-C, Kim G-T (2002) The clinical effect on low back pain by acupuncture treatment and GCM(General Coordinative Manipulation) treatment. J Oriental Rehab Med 12: 111-120.

12. Ryu S-M, Hwang J-S, Rhu E-K (2001) The Clinical Effect on Low Back Pain by Young-gol and Dae-back Acupuncture Points. J Oriental Rehab Med 12: 1-10.

13. Kim K-T, Song H-S (2005) The Effectiveness of Bee Venom Acupuncture Therapy on the Treatment of Sprain of L-spine(A Randomized Controlled Trial; Double Blinding). J Kor Acup Mox Soc 22: 113-120.

14. Yoon K-S, Lee H, Kang J-H, Choi, J-Y (2010) Comparison Study on 30 Cases of HIVD Patients with Restricted on SLRT by Sa-Am Acupuncture Banggwangjeonggyeok (膀胱正格) and General Acupuncture. J Kor Acup Mox Soc 27: 79-87.

15. Lee J-W (2006) A Randomized Controlled Trial of Acupuncture Effect of Kuesu-point(快兪穴) on Lower back pain and sciatica [LBP_MS062]: Dongguk University.

16. Lee SH, Kim SK, Woo NS, Lee YC, Chang SK, et al. (1996) The Effect of Acupuncture-like TENS on Finger Control Gate - Patients with cervical sprain and postoperative pain of laminectomy-. J Korean Pain Soc 9: 140-144.

17. Lee J-S, Song K-H, Lee S-N, Kim D-J, Yu J-S, et al. (2007) Clinical Study of Different Effect between Trigger Point N eedling and Remote Acupuncture Point Needling on Shoulder Pain Patient. J Kor Acup Mox Soc 24: 89-96.

18. Nam D-W, Kim H-B, Yang D-H, Lim S, Kim K-S, et al. (2006) Comparison Research of Clinical Effect of Eastern and Western Medical Treatment on Frozen Shoulder Patients. J Kor Acup Mox Soc 23: 105-113.

19. Kim C-Y, Kwon N-H, Shin Y-J, Nam D-W, Kim K-H, et al. (2007) Randomized Controlled Trial : Effect of Master Dong s Acupuncture in Chronic Shoulder Pain Patients. J Kor Acup Mox Soc 24: 89-96.

20. Nam D-W, Jung I-T, Kim J-H, Park Y-S, Lim S, et al. (2006) Clinical Observation of Western Medical Treatment and Acupuncture Treatment on Frozen Shoulder Patients. J Kor Acup Mox Soc 23: 177-185.

21. Sok SH, Kim KB (2000) The Effect of Auricular Acupuncture Pressure Therapy on Insomnia of Elderly People. Kor J Adult Nurs 12: 222-233.

22. Kim Y-S, Kim C-H, Kim K-S (1996) Effect of Auricular Acupuncture on Postoperative Nausea and Vomiting. Kor J Oriental Med 17: 331-336.

23. Lee SK, Lee SW, Choi DH (2007) Effect of Acupuncture on P6 for Preventing Opioid-induced Nasea and Vomiting. Korean J Oriental Physiology & Pathology 21: 1637-1640.

24. Hong K-E, Park Y-C, Jo J-H, Jo H-G, Jeong I-C, et al. (2007) Effect of Sa-am Acupuncture Method for Chronic Tension-type Headache: A Randomized Controlled Trial. J Kor Acup Mox Soc 24: 13-28.

25. Lee S-H, Hong S-J, Kim S-Y, Yang H-I, Lee J-D, et al. (2003) Randomized Controlled Double Blind Study of Bee Venom Therapy on Rheumatoid Arthritis. J Kor Acup Mox Soc 20: 80-88.

26. Joung W-J, Wang K-H, Kim K-H, Bae J-I, Kim S-H, et al. (2010) The Effect of Acupuncture at Fengchi(GB20) and Houxi(SI3) for Acute Headache Due to Whiplash Injury－Randomised Clinical Trial－. J Kor Acup Mox Soc 27: 127-135.

27. Song B-Y (2007) A Clinical Study on the Effects of Sweet Bee Venom Herbal Acupuncture for Patients with Whiplash Injury. J Pharmacopuncture 10: 77-83.

28. Seol H, Sin M-S, Song B-Y, Yook T-H (2005) Effects of Jungsongouhyul Herbal Acupuncture(JSO) Multi-treatment for Whiplash Injury by Traffic Accident. J Pharmacopuncture 8: 59-65.

29. Cho J-H, Chung S-H, Kim S-S (2006) The Effect of Dong-Si Acupoint on the Meridian Muscle Tension of Governer Vessel and Bladder Meridian. J Oriental Rehab Med 16: 82-92.

30. Park J-Y, Yun K-J, Choi Y-J, Kim M-S, Jeon J-C, et al. (2011) Comparative Study of Treatment Effect between Near Acupuncture Point Needling and Near Acupuncture with Remote Acupuncture Point Needling on Treatment of Posterior Neck Pain. J Kor Acup Mox Soc 28: 85-92.

31. Lee K-H, Youn H-M, Ko W-S, Song C-H, Jang K-J, et al. (2008) Comparison of Treatment Effects and Allergic responses to stiff neck between Sweet Bee Venom and Bee Venom Pharmacopuncture (A pilot study, Double blind, Randomized Controlled Clinical Trail). J Pharmacopuncture 11: 39-48.

32. Kim K-T, Song H-S (2005) A Randomized Control1ed Double Blinding Study of Bee Venom Acupuncture Therapy on Sprain of C-spine. J Kor Acup Mox Soc 22: 189-195.

33. Kang Y-H, Kim H-E, Cho M-J, Kim T-W, Yoon K-B, et al. (2002) The Clinical Effects of Korean Bee-Venom Therapy in Neck Pain Due to Soft Tissue Damage. J Kor Acup Mox Soc 19: 67-79.

34. Kim H-E, Kang Y-H, Cho M-J, Kim T-W, Kim E-Y, et al. (2002) The Clinical Effects of Carthami-Flos Herbal Acupuncture in Neck Pain Due to Soft Tissue Damage. J Kor Acup Mox Soc 19.

35. Lee M-K (2003) Effects of San-Yin-Jiao(SP6) Acupressure on Labor Pain, Delivery Time in Women during Labor. J Korean Acad Nurs 33: 753-761.

36. Cho J-H (2007) A Pilot Study of the Difference between Gyeiibongnyeong-hwan and Gyejibongnyeong-hwan combined Acupuncture Therapy on the Primary Dysmenorrhea. J Oriental Obsterics & Gynecology 20: 161-168.

37. Han M-S (2009) Effectof 5 Element Acupuncture Treatment on the Primary Dysmenorrhea [Dysmenorrhea_OBGY005]: Dong Eui University.

38. Kim S-M, Jang S-H, Kim C-H, Youn H-M, Song C-H, et al. (2008) Effect of Hominis placenta Pharmacopuncture on the Dysmenorrhea (A Pilot study, Single blind, Randomized, Controlled Clinical Trial). J Pharmacopuncture 11: 123-131.

39. Lee I-S, Youn H-M, Jung K-K, Kim S-M, Min Y-K, et al. (2007) Effect of Sa-am Acupuncture Treatment on the Dysmenorrhea (Pillot Study, Single Blind, Randomized, Sham Acupuncture, Controlled Clinical TriaI). J Kor Acup Mox Soc 24: 63-79.

40. Yuk S-S, Lim E-M (2005) A Clinical Study on the Effect of Crossing over Treatment of Acupuncture and Herbal Medication for Primary Dysmenorrhea. J Oriental Obsterics & Gynecology 18: 144-152.

41. Lee HJ, Kim KS, Koo SL (2005) Effects of Capsicum Plaster at the Korean Hand Acupuncture Point on Pain Management after Knee Replacement. Korean J Anesthesiol 48: 398-402.

42. Na WM, Lee SY, Jang EH, Kim SC, Moon HC, et al. (2007) A Study on Pain relief effects and Allergic responses for the Osteoarthritis of the knee joint Between Sweet Bee Venom and Bee Venom Pharmacopuncture. J Pharmacopuncture 10: 47-55.

43. Min M-H, Choi Y-G, Kim Y-J, Park H-J, Lee S-C, et al. (2009) The Effect of Sa-am acupuncture on Knee Osteoarthritis. Kor J Meridian Acupoint 26: 53-66.

44. Ryu S-M, Lee J-S, Kim, S-S, Jung S-H (2004) The Effect of Intra-articular Bee Venom Injection on Osteoarthritis of the Knee. J Oriental Rehab Med 14.

45. Kim S-C, Lim J-A, Lee J-D, Lee S-K, Lee S-Y, et al. (2006) A Pilot Study of Acupuncture Treatment for the Osteoarthritis of the Knee J oint on the EBM (Evidence Basement Medicine). J Kor Acup Mox Soc 23: 187-215.

46. Lee S-N, Hong S-Y, Jo H-C, Byun I-J, Song H-S, et al. (2003) The Clinical Study on Bee Venom Acupuncture Treatment on Osteoarthritis of Knee Joint. J Kor Acup Mox Soc 20: 73-81.

47. Seo S-K, Cho W-S, Lee J-W, Kim Y-N, Jung J-K, et al. (2008) Effects of Auricle Electric Stimulation on Pain, Gait and Balance in the Old Aged with Knee Joint Disease. J Kor Soc Phys Ther 20: 11-17.

48. Park K-B, Song K-H, Lee J-S, Jo J-H (2006) Study on Clinical Effects of Homnis Placenta Herbal Acupuncture on Osteoarthritis of Knee J oint. J Kor Acup Mox Soc 23: 163-173.

49. Kim EJ (2009) Efficacy of Root bark of Ulmus davidiana Planch Pharmacopuncture on the Patients with Knee Osteoarthritis : Double Blinded Randomized Controlled Trial [Peripheral OA_MS025]: Dongguk University.

50. Kim H-B, Lee R-M, Lee M-H, Choi Y-S, Kim J-I, et al. (2008) Comparative Study of Effects of "Intramuscular Bee Venom Herbal Acupuncture' and "Intracutaneous Bee Venom Herbal Acupuncture' in Knee Osteoarthritis Patients. J Kor Acup Mox Soc 25: 151-164.

51. Park I-S, Jung C-Y, Jang M-K, Kang M-S, Lee S-W, et al. (2008) A Randomized Clinical Trial of Local Acupoints Compared with Distal Acupoints in Degenerative Osteoarthri tis on Knee. J Kor Acup Mox Soc 25: 227-242.

52. Byun H, Kim S-W, Ahn J-H, Kim Y-S, Seo J-C, et al. (2007) Individualized Acupuncture versus Standardized Acupuncture in Symptomatic Treatment of Osteoarthritis of the Knee-a Randomized Controlled Trial (ISRCTN 40706107). J Kor Acup Mox Soc 24: 183-195.

53. Min WK (2008) A Comparative study of Warm needling and Acupuncture on Osteoarthritis of the Knee - a Randomized Controlled Trial -. Kyung Hee University.

54. Chae S-J, Song H-S (2004) The Effect of 8 Constitution Acupuncture on degenerative arthritis of knee joint. J Kor Acup Mox Soc 21: 65-73.

55. Yang KR, Song HS (2008) A Comparative study of Warm needling and Bee Venom Pharmacopuncture on Osteoarthritis of the Knee - a Randomized Controlled Trial -. J Pharmacopuncture 11: 21-31.

56. An B-J, Kim K-T, Kang M-S, Song H-S (2006) Effect of Bee Venom-acupuncture on Patients with Osteoarthritis of Knee Joint. J Kor Acup Mox Soc 23: 15-20.

57. An G-H, Lee H, Lee B-R (2004) The Comparative Study on the Bee-Venom Therapy and Common Acupuncture Therapy for the Latera1 Epicondylitis (Tennis Elbow). J Daejeon Oriental Medicine 13: 267-276.

58. Chae YB, Lee JC, Park KM, Lee HJ, Kang OS, et al. (2009) Inhibitory effect of acupuncture at HT7 on the sympathetic activations to smoking-related visual cues during smoking cessation. The Korean Journal of Meridian & Acupoint 26: 39-52.

59. Kim M-S, Kim H-J, Park Y-J, Kim E-H, Lee E-Y (2004) The clinical research of the efficacy of bee venom aqua-acupuncture on peripheral facial paralysis. J Kor Acup Mox Soc 21: 251-262.

60. Kim N-O, Chae S-J, Son S-S (2001) Comparative Clinical Study between Oriental Medicine and Oriental-Western Medicine Treatment on Bell's palsy. J Kor Acup Mox Soc 18: 99-108.

61. Kim S-H, Kim J-S, Lee B-H, Lim S-C, Jung T-Y, et al. (2010) Comparative Clinical Study of Jung-an Acupuncture and General Acupuncture on Bell's Palsy Patients. J Kor Acup Mox Soc 27: 43-49.

62. Lee C-W, Park I-B, Kim S-W, Kim H-G, Heo S-W, et al. (2004) The Effect of Acupuncture and Dong's Acupuncture about Bell's palsy. J Kor Acup Mox Soc 21: 287-300.

63. Park J-H, Jang S-H, Lee C-H, Ku J-Y, Jeun D-S, et al. (2010) The Clinical Research of the Effectiveness of Pharmacopuncture Complex Therapy on Peripheral Facial Paralysis - Hominis Placenta Pharmacopuncture Therapy and Sweet Bee Venom Therapy －. J Kor Acup Mox Soc 27: 79-87.

64. Choi Y-J, Yoon K-J, Kim M-S, Park J-Y, Jeon J-C, et al. (2010) Effects of Scalp Acupuncture with Usual Acupuncture on Peripheral Facial Palsy in Comparison with Usual Acupuncture Only. J Kor Acup Mox Soc 27: 101-109.

65. Shin H-W, Kang J-H, Lee H (2009) Efficacy of Soyeom Pharmacopuncture on Postauricular Pain Accompanied with Peripheral Facial Paralysis. J Kor Acup Mox Soc 26: 41-49.

66. An B-J, Song H-S (2005) Effect of Electroacupuncture on Patients with Peripheral Facial Paralysis. J Kor Acup Mox Soc 22: 121-129.

67. Chun H-S, Lee J-E, Cho M, Ryu C-R, Ryu M-S, et al. (2009) Effects of Selection Method of Acupuncture between the Affected Part and the Unaffected Part on Peripheral Facial Nerve Paralysis in the Incipient Stage. J Kor Acup Mox Soc 26: 179-186.

68. Choi B-C, Han K-S, Ahn T-W (2009) Clinical comparison studies on 30 cases of Bell's palsy patient with postauricular pain by Anti-inflammatory pharmacopuncture & Acupuncture and Herbal therapy. J Daejeon Oriental Medicine 18: 89-94.

69. Choi J-Y, Lee H, Kang J-H, Kim Y-I, Kim J-H, et al. (2009) Comparative Study of General Oriental Medical Treatment and Bee Venom Pharmacopuncture on Acute Peripheral Facial Paralysis Patient with Postauricular Pain. J Kor Acup Mox Soc 26: 95-103.

70. Hwang Y-J, Lee H, Heo Y-K, Song H-G, Ahn T-W, et al. (2006) Comparison studies on 20 cases of Bell's palsy patients by acupunture and Rainbow therapy & acupunture. J Daejeon Oriental Medicine 15: 87-95.

71. Lee S-W, Yun J-M, Son J-W, Kang B-G, Park S-M, et al. (2007) The Effect of Electroacupuncture on Upper-Extremity Spasticity of Stroke Patients. Korean J Orient Int Med 28: 492-501.

72. Lee H-S, Lee J-S, Kim S-S (2005) The Comparison of Effectiveness in Electroacupuncture between Dong-si Acupoint and Body Acupoint in Hemiplegic Patients after Acute Stroke. J Oriental Rehab Med 15: 55-64.

73. Cho J-H, Chung S-H, Lee J-S, Kim, S-S (2006) MEMG Analysis on Antispastic Effect of Electroacupuncture and Transcutaneous Electrical Nerve Stimulation. J Oriental Rehab Med 16: 131-143.

74. Cho S-W, Go K-H, Nam J-H, Kim M-S, Lee S-Y, et al. (2005) The Effectiveness of Zingiberis Rhizoma Herbal Acupuncture Therapy and Bee Venom Herbal Acupuncture Therapy on the Poststroke Hemiplegic Shoulder Pain. J Oriental Rehab Med 15: 77-87.

75. Kim M-B, Shin H-D, Kim S-S (2005) The Influences of Electroacupuncture at Interosseous Muscle for Hand Function in Hemiplegic Patients after Stroke. J Oriental Rehab Med 15: 17-28.

76. Lee D-Y, Lee G-M, Yeom S-C, Kim D-H, Kim D-J (2006) A Clinical Study of Bee Venom Acupuncture Therapy on Shoulder Pain Patients in Stroke Sequelae. J Kor Acup Mox Soc 23: 69-80.

77. Seo J-C, Chung W-J, Kim J-H, Nam S-S, Lee J-D, et al. (1998) The Effect of Cranial and Penetrational Acupuncture on Recovery of Motor Disorder due to Stroke. J Korean Oriental Med 3: 19-27.

78. Eom JY, Won SH, Kwon KR, Lee HS (2009) Comparative study of Acupuncture, Bee Venom Acupuncture and Bee Venom Herbal Acupuncture on the treatment of Post-stroke Hemiplegic Shoulder Pain. J Pharmacopuncture 9: 139-154.

79. Ko C-N, Min I-K, Park S-W, Jung W-S, Moon, S-K, et al. (2007) Effectiveness of Bee Venom Acupuncture on Shoulder Pain after Stroke. J Korean Oriental Med 28: 11-24.

80. Choi DY, Lee DI, Klm SY, Klm KS, Lee JD, et al. (2003) Speciflc Electric Acupuncture for the Management of the Central Poststroke Pain. EAST-WEST MEDICAL RESEARCH INSTITUTE JOURNAL 2003: 171-177.

81. Lee SH, Lee YH (1997) Clinical Study with Thermography on Shoulder Hand Syndrome after Stroke. J Korean Oriental Med 18: 25-39.

82. Cho T-S, Son I-S, Kim C-H, Seo J-C, Youn H-M, et al. (2002) Effects of Added Tong's Acupuncture on NIH Stroke Scale and MBI in Stroke Patients. J Kor Acup Mox Soc 20: 35-45.

83. Ryu H-J, Kim S-S (2006) Effect of Electroacupuncture by Different Insertion Method on Upper Limb Function in Post Stroke Patients with Hemiplegia. J Oriental Rehab Med 16: 49-61.

84. Bae H-H, Park Y-C (2004) Effect of Joongseungerhyul Herbal Acupuncture on Function and Quality of Life in Patients with Poststroke Shoulder Pain (a Randomized Double Blind Study). J Pharmacopuncture 7: 77-86.

85. Sim W-J, Jung S-H, Kim S-S, Shin H-D, Lee J-S (2003) Which is More Effective for Elbow Spasticity after Stroke, the Electroacupuncture on Yin or Yang Meridians? J Oriental Rehab Med 13: 95-111.

86. Kim J-Y, Jeong S-M, Park C-K, Min E-K, Wang T-C (2008) The Clinical Effectiveness of Acupuncture at Palsa(BaXie) for Hand Function in Hemiparetic Patients after Stroke. J Kor Acup Mox Soc 25: 97-104.

87. Noh J-H, Park J-A, Cho S-W, Youn H-M, Jang K-J, et al. (2010) Effect of Bee-venom Acupuncture on Upper Limb Spasticity of Stroke Patients. J Kor Acup Mox Soc 27: 115-125.

88. Ryu SH (2001) Effects of Electroacupuncture Stimulation on the Hemiplegic Upper Extremity after Stroke. Kyung Hee University.

89. Noh J-H, Park J-A, Youn H-M, Jang K-J, Song C-H, et al. (2009) The effect of Hominis Placenta Pharmacopuncture on Leg spasticity of stroke patients (A Pilot study, Double blind, Randomized, Controlled Clinical Trial). J Pharmacopuncture 12: 97-119.

90. Gwak J-Y, Cho S-Y, Shin A-S, Lee I-W, Kim N-H, et al. (2009) Efficacy of Bee-venom Acupuncture on Central Post Stroke Pain－Single-blind Randomized Controlled Trial－. J Kor Acup Mox Soc 26: 205-214.

91. Kim Y-S (2000) Antispastic Effects of Electroacupuncture, TENS and NMES in Stroke Patient. J Kor Acup Mox Soc 17: 209-220.

92. Lee S-H, Kim E-J, Yun S-P, Kim L-D, Lee C-R, et al. (2004) The Effect of Intradermal Acupuncture on The Patients suffering from Insomnia after Stroke. Korean J Orient Int Med 25: 138-148.

93. Cho T-S, Son I-S, Park I-B, Kim S-W, Seo J-C, et al. (2003) Effects of Scalp Acupuncture on Short-term NIHSS and MBI in Stroke Patients. J Korean Oriental Med 24: 65-73.

94. Seo J-C, Cheong B-S, Yun H-S, Cho S-G, Kim Y-M, et al. (2001) Effects of Contralateral Acupuncture on Recovery of Motor Disorders in Stroke Patients. J Kor Acup Mox Soc 18: 1-9.

95. Seo J-C, Baek Y-H, Nam T-H, Seo D-M, Lee H-J, et al. (2001) Effects of Contralateral Both Side Acupuncture on NllI Scale in Stroke Patients. J Kor Acup Mox Soc 22: 98-104.

96. Kim Y-S, Hong J-W, Jung W-S, Na B-J, Park S-U, et al. (2007) A Comparative Study of Motor Recovery from Stroke between High and Low Frequency Electrical Acupoint Stimulation. J Korean Oriental Med 28: 289-298.

97. Ryu S-H, Lee K-S, Kim T-K, Choi Y-S, Yun S-P, et al. (2002) Effects of Electroacupuncture on the Hemiplegic Upper Extremity after Stroke. J Korean Oriental Med 23: 180-189.

98. Yang C-Y, Shin B-C, Chong B-H (2008) The Effect of Double Application of Functional Electrical Stimulation in Patients with Dysphgia after Stroke. J Oriental Rehab Med 18: 111-123.

99. Seong YS, Cho E-Y, Lee Y-S, Yang HL, Lee HJ (2010) Effects of Nei-Guan Acupressure on Chemotherapy-related Nausea, Vomiting, and Anorexia in Patients with Lung Cancer. J East-West Nurs Research 16: 1-10.
