# Supplementary material for: Impact of Including Korean Randomized Controlled Trials in Cochrane Reviews of Acupuncture
Source: PLoS One. 2012 Oct 11;7(10):e47619. doi: 10.1371/journal.pone.0047619 (PMC3469498; doi:10.1371/journal.pone.0047619)
Supplement: Table S3 — Seven new meta-analyses after the inclusion of Korean trials. (DOC) [file pone.0047619.s003.doc]

| Table S3. Seven new meta-analyses after the inclusion of Korean trials | | | | | |
| --- | --- | --- | --- | --- | --- |
| Review topics | Comparisons | Outcomes | Effect estimates before inclusion | Number of Korean studies | Effect estimates after inclusion |
| Dysmenorrhea | Acupuncture vs placebo | Pain relief, short term | -0.23 [-0.64, 0.18] | 1 | -0.14 [-0.47, 0.20] I^2^=0% |
| Insomnia | Acupuncture vs sham acupuncture | Post-treatment sleep quality score | -0.29 [-0.77, 0.20] | 1 | Not estimable |
| LBP | Acupuncture vs acupuncture (subacute LBP < 3months) | Pain measured by VAS: immediately after the end of the sessions | no study before the inclusion | 2 | -0.47 [-0.91, -0.02] I^2^=0% |
|  | Acupuncture vs placebo (Chronic LBP>3months) | Pain disability index: immediately after the end of sessions | -0.35 [-0.81, 0.11] | 1 | -0.18 [-0.57, 0.20] I^2^=15% |
|  | Acupuncture vs acupuncture (unknown/mixed duration of LBP) | Pain: immediately after the end of sessions | 0.46 [-0.48, 1.40] | 1 | 0.03 [-0.45, 0.52] I^2^=8% |
|  | Acupuncture plus intervention versus other intervention alone (unknown/mixed duration of LBP) | Functional status: immediately after the end of sessions | -0.38 [-1.31, 0.55] | 1 | -0.27 [-0.71, 0.18] I^2^=0% |
| PONV | P6 stimulation vs sham | Rescue antiemetics: Droperidol | 0.71 [0.22, 2.28] | 1 | 1.03 [0.43, 2.47] I^2^=0% |

PONV: postoperative nausea and vomiting
