# Supplementary material for: Impact of Including Korean Randomized Controlled Trials in Cochrane Reviews of Acupuncture
Source: PLoS One. 2012 Oct 11;7(10):e47619. doi: 10.1371/journal.pone.0047619 (PMC3469498; doi:10.1371/journal.pone.0047619)
Supplement: Table S4 — 24 augmented meta-analyses after the inclusion of Korean trials. (DOC) [file pone.0047619.s004.doc]

| Table S4. 24 augmented meta-analyses after the inclusion of Korean trials | | | | | | |
| --- | --- | --- | --- | --- | --- | --- |
|  |  |  | Before inclusion | | After inclusion | |
| Review topics | Comparisons | Outcomes | Number of studies | Effect estimates | Number of studies | Effect estimates |
| Insomnia | Acupuncture vs no treatment | Post-treatment sleep quality score | 2 | -0.55 [-0.89,-0.21] I^2^=0% | 3 | -1.69[-3.23,-0.14] I^2^=95% |
| Tension-type headache | Acupuncture vs sham interventions | Headache intensity: up to 8 weeks/2 months later | 2 | -0.16 [-0.53,0.21] I^2^=0% | 3 | -0.12 [-0.45,0.21] I^2^=0% |
|  |  | Headache intensity: 3 to 4 months after randomization | 3 | -0.12 [-0.28,0.04] I^2^=0% | 4 | -0.10 [-0.25,0.06] I^2^=0% |
| LBP | Acupuncture vs placebo (Chronic LBP>3months) | Pain: immediately after the end of treatment | 4 | -0.47 [-0.69,-0.24] I^2^=0% | 5 | -0.43 [-0.63,-0.22] I^2^=0% |
|  |  | Side effects / Complications: immediately after the end of the sessions | 2 | Not applicable^*^ | 3 | Not applicable^*^ |
|  | Acupuncture plus intervention versus other intervention alone (Chronic LBP>3months) | Immediately after the end of sessions | 4 | -0.76[-1.02,-0.50] I^2^=14% | 5 | -0.75 [-0.98,-0.52] I^2^=0% |
| PONV |  | Nausea (all trials) | 27 | 0.71 [0.61,0.83] I^2^=60% | 29 | 0.71 [0.61,0.83] I^2^=59 |
|  |  | Nausea (unclear or inadequate sequence generation) | 11 | 0.77 [0.61,0.97] I^2^=37% | 13 | 0.76 [0.61,0.94] I^2^=37 |
|  |  | Nausea (unclear or inadequate allocation concealment) | 23 | 0.70 [0.59,0.84] I^2^=56% | 25 | 0.70 [0.59,0.83] I^2^=55% |
|  |  | Nausea (adequate blinding of outcome assessor) | 23 | 0.69 [0.58,0.82] I^2^=63% | 25 | 0.69 [0.58,0.81] I^2^=61% |
|  |  | Nausea (Unclear or not free of selective reporting) | 8 | 0.78 [0.59,1.02] I^2^=37% | 10 | 0.76 [0.59,0.98] I^2^=37% |
|  |  | Nausea (trials with control event rate more than 20%) | 24 | 0.71 [0.60,0.83] I^2^=64% | 26 | 0.70 [0.60,0.82] I^2^=63% |
|  |  | Nausea (adults) | 24 | 0.74 [0.63,0.88] I^2^=59% | 26 | 0.74 [0.63,0.87] I^2^=58% |
|  |  | Nausea (noninvasive P6 stimulation) | 21 | 0.73 [0.61,0.88] I^2^=60% | 23 | 0.73 [0.61,0.87] I^2^=59% |
|  |  | Vomiting (all trials) | 32 | 0.70 [0.59,0.83] I^2^=53% | 34 | 0.69 [0.59,0.82] I^2^=52% |
|  |  | Vomiting (unclear or inadequate sequence generation) | 16 | 0.68 [0.53,0.87] I^2^=62% | 18 | 0.66 [0.52,0.84] I^2^=60% |
|  |  | Vomiting (unclear or inadequate allocation concealment) | 28 | 0.69 [0.57,0.84] I^2^=58% | 30 | 0.68 [0.56,0.82] I^2^=57% |
|  |  | Vomiting (adequate blinding of outcome assessor) | 26 | 0.70 [0.58,0.84] I^2^=58% | 28 | 0.69 [0.57,0.83] I^2^=57% |
|  |  | Vomiting (Unclear or not free of selective reporting) | 12 | 0.68 [0.52,0.89] I^2^=63% | 14 | 0.66 [0.51,0.86] I^2^=60% |
|  |  | Vomiting (trials with control event rate less than or equal to 20%) | 9 | 0.86 [0.57,1.29] I^2^=4% | 10 | 0.83 [0.53,1.29] I^2^=12% |
|  |  | Vomiting (trials with control event rate more than 20%) | 23 | 0.67 [0.56,0.81] I^2^=62% | 24 | 0.67 [0.56,0.80] I^2^=61% |
|  |  | Vomiting (adults) | 25 | 0.73 [0.62,0.86] I^2^=20% | 27 | 0.72 [0.61,0.84] I^2^=19% |
|  |  | Vomiting (noninvasive P6 stimulation) | 25 | 0.71 [0.58,0.86] I^2^=54% | 27 | 0.69 [0.57,0.85] I^2^=53% |
|  |  | Rescue antiemetics (Ondancetron) | 10 | 0.68 [0.52,0.89] I^2^=40% | 11 | 0.68 [0.52,0.89] I^2^=40% |

LBP: low back pain

PONV: postoperative nausea and vomiting

*: This outcome had no summed effect estimate.
