# Supplementary material for: Impact of Including Korean Randomized Controlled Trials in Cochrane Reviews of Acupuncture
Source: PLoS One. 2012 Oct 11;7(10):e47619. doi: 10.1371/journal.pone.0047619 (PMC3469498; doi:10.1371/journal.pone.0047619)
Supplement: Table S5 — 50 Single-study forest plots generated by the inclusion of Korean studies. (DOC) [file pone.0047619.s005.doc]

| Table S5. 50 Single-study forest plots generated by the inclusion of Korean studies | | | |
| --- | --- | --- | --- |
| Review topics | Comparisons | Outcomes | Effect estimates |
| Neck disorders | Acupuncture versus waitlist control | Pain intensity (VAS) post-treatment | -1.13 [-1.78, -0.48] |
| Shoulder pain | Local and distal acupuncture versus local acupuncture points (adhesive capsulitis) | Pain post-intervention: VAS | -0.09 [-0.61, 0.43] |
|  |  | Pain post-intervention: pain scores in SPADI | -0.06 [-0.59, 0.46] |
|  |  | Shoulder discomfort: disability scores in SPADI | -0.03 [-0.56, 0.49] |
|  |  | Total SPADI scores | -0.05 [-0.57, 0.48] |
|  |  | Range of motion: External rotation | 0.15 [-0.38, 0.67] |
|  |  | Range of motion: Adduction | -0.02 [-0.54,0.51] |
|  | Acupuncture versus placebo (Adhesive capsulitis) | Pain post-intervention: VAS | 0.00 [-0.45,0.45] |
|  |  | Pain post-intervention: pain scores in SPADI | -0.04 [-0.49, 0.41] |
|  |  | Range of adduction | 0.04 [-0.41, 0.49] |
|  |  | Range of external rotation | 0.28 [-0.17, 0.73] |
|  |  | Shoulder discomfort: disability scores in SPADI | 0.01 [-0.43, 0.46] |
|  |  | Total SPADI scores | -0.01 [-0.46, 0.44] |
|  | Acupuncture plus suprascapular nerve block, subacromial steroid injection, and trigger point injection versus acupuncture (Adhesive capsulitis) | Pain postintervention: VAS | -1.11 [-1.82, -0.40] |
|  |  | Overall Success (Constant Murley score) at week 4 | -0.45 [-1.11, 0.21] |
|  |  | Total SPADI scores at week 4 | -0.38 [-1.04, 0.28] |
|  |  | Range of abduction | -0.60 [-1.27, 0.07] |
|  |  | Range of adduction | -0.43 [-1.10, 0.23] |
|  |  | Range of flextion | -0.34 [-1.00, 0.32] |
|  |  | Range of extension | -0.40 [-1.06, 0.26] |
|  | Acupuncture plus suprascapular nerve block, subacromial steroid injection, and trigger point injection versus acupuncture (General shoulder pain: no diagnosis given) | Pain postintervention: VAS | -0.27 [-0.93, 0.39] |
|  |  | Overall Success (Constant Murley score) at week 4 | -0.60 [-1.27, 0.07] |
|  |  | Total SPADI scores at week 4 | -0.13 [-0.79, 0.52] |
|  | Acupuncture plus suprascapular nerve block, subacromial steroid injection, and trigger point injection versus suprascapular nerve block, subacromial steroid injection, and trigger point injection (Adhesive capsulitis) | Pain postintervention: VAS | 0.04 [-0.65, 0.72] |
|  |  | Overall Success (Constant Murley score) at week 4 | -0.15 [-0.84, 0.53] |
|  |  | Total SPADI scores at week 4 | -0.19 [-0.87, 0.50] |
|  |  | Range of abduction | -0.16 [-0.85, 0.52] |
|  |  | Range of adduction | -0.32 [-1.01, 0.37] |
|  |  | Range of flextion | -0.14 [-0.83, 0.54] |
|  |  | Range of extension | -0.52 [-1.22, 0.18] |
|  | Acupuncture plus suprascapular nerve block, subacromial steroid injection, and trigger point injection versus waitlist (General shoulder pain: no diagnosis given) | Pain postintervention: VAS | -1.02 [-1.72, -0.32] |
|  |  | Overall Success (Constant Murley score) at week 4 | -1.04 [-1.74, -0.34] |
|  |  | Total SPADI scores at week 4 | -0.35 [-1.01, 0.31] |
|  | Acupuncture versus waitlist (General shoulder pain: no diagnosis given) | Pain postintervention: VAS | -0.32 [-0.98, 0.34] |
|  |  | Overall Success (Constant Murley score) at week 4 | -0.39 [-1.05, 0.27] |
|  |  | Total SPADI scores at week 4 | -0.21 [-0.87, 0.45] |
|  | Acupuncture versus suprascapular nerve block, subacromial steroid injection, and trigger point injection (Adhesive capsulitis) | Pain Post Intervention: VAS | 1.00 [0.27, 1.73] |
|  |  | Overall Success (Constant Murley score) at week 4 | 0.24 [-0.45, 0.93] |
|  |  | Total SPADI scores at week 4 | 0.12 [-0.57, 0.80] |
|  |  | Range of abduction | 0.41 [-0.28, 1.10] |
|  |  | Range of adduction | 0.16 [-0.53, 0.85] |
|  |  | Range of flexion | 0.16 [-0.53, 0.84] |
|  |  | Range of extension | -0.14 [0.83, 0.54] |
| Cancer pain in adults | Bee venom acupuncture versus placebo | Average pain measured at each immediate post-treatment | -2.65 [-4.48, -0.83] |
|  |  | Pain in last 24 hours | -0.23 [-1.55, 1.10] |
| Insomnia | Acupressure versus no treatment | Self-rated improvement in sleep quality | -9.22 [-11.44, -7.01] |
| Low back pain | Acupuncture versus acupuncture. ((Sub)acute LBP: < 3 months) | Range of motion (flexion): Immediately after the end of the sessions | -0.01 [-0.56, 0.55] |
|  |  | Range of motion (extension): Immediately after the end of the sessions | -0.30 [-0.86, 0.26] |
|  |  | Functional status (standardized measures): Immediately after the end of the sessions | -0.98 [-1.76, -0.20] |
|  | Acupuncture versus placebo or sham intervention (Chronic LBP: > 3 months) | Functional status (standardized measures): Immediately after the end of the sessions | 0.05 [-0.51, 0.60] |

VAS: visual analogue scale

SPADI: shoulder pain and disability index

LBP: low back pain
